# Alevin-fry-atac enables rapid and memory frugal mapping of single-cell ATAC-seq data using virtual colors for accurate genomic pseudoalignment

**DOI:** 10.1093/bioinformatics/btaf234

**Published:** 2025-07-15

**Authors:** Noor Pratap Singh, Jamshed Khan, Rob Patro

**Affiliations:** Department of Computer Science, University of Maryland, College Park, MD 20742, United States; Department of Computer Science, University of Maryland, College Park, MD 20742, United States; Department of Computer Science, University of Maryland, College Park, MD 20742, United States

## Abstract

**Summary:**

Ultrafast mapping of short reads via lightweight mapping techniques such as pseudoalignment has significantly accelerated transcriptomic and metagenomic analyses with minimal accuracy loss compared to alignment-based methods. However, applying pseudoalignment to large genomic references, like chromosomes, is challenging due to their size and repetitive sequences. We introduce a new and modified pseudoalignment scheme that partitions each reference into “virtual colors.” These are essentially overlapping bins of fixed maximal extent on the reference sequences that are treated as distinct “colors” from the perspective of the pseudoalignment algorithm. We apply this modified pseudoalignment procedure to process and map single-cell ATAC-seq data in our new tool alevin-fry-atac. We compare alevin-fry-atac to both Chromap and Cell Ranger ATAC. Alevin-fry-atac is highly scalable and, when using 32 threads, is 2.8 times faster than Chromap (the second fastest approach) while using only 33% of the memory required by Chromap. The resulting peaks and clusters generated from alevin-fry-atac show high concordance with those obtained from both Chromap and the Cell Ranger ATAC pipeline, demonstrating that virtual color-enhanced pseudoalignment directly to the genome provides a fast, memory-frugal, and accurate alternative to existing approaches for single-cell ATAC-seq processing. The development of alevin-fry-atac brings single-cell ATAC-seq processing into a unified ecosystem with single-cell RNA-seq processing (via alevin-fry) to work toward providing a truly open alternative to many of the varied capabilities of CellRanger.

**Availability and implementation:**

Alevin-fry-atac is written in Rust and C++17, and is freely-available under a BSD 3-clause license. It is integrated into piscem (https://github.com/COMBINE-lab/piscem) and alevin-fry (https://github.com/COMBINE-lab/alevin-fry), and is also supported directly as part of simpleaf (https://github.com/COMBINE-lab/simpleaf)

## 1 Introduction

ATAC-seq (Buenrostro *et al.* 2013) is a widely used assay that enables the profiling of open-chromatin regions within the genome. It has a wide variety of applications, such as providing insight into gene regulation through the identification of promoters and enhancers (Starks *et al.* 2019), studying cell differentiation and development (Lara-Astiaso *et al.* 2014, Preissl *et al.* 2018), identification of transcription factors (Schep *et al.* 2015) to name a few. As with RNA-seq, it is also now possible to profile chromatin at the level of single cells (Buenrostro *et al.* 2015, Cusanovich *et al.* 2015). However, the efficient mapping and processing of single-cell ATAC-seq is a computationally challenging task.

Currently, three main pipelines/methods are used for this task. One involves aligning reads to the genome using methods such as BWA-MEM (Li and Durbin 2009), or Bowtie2 (Langmead and Salzberg 2012), followed by demultiplexing into cells and then finally using existing general-purpose programs like SAMtools (Li *et al.* 2009) and Picard (Picard Toolkit 2019) to sort and filter the alignments. This pipeline can be quite slow when working with large datasets and can be sped up to some extent [e.g. by replacing one of the above alignment tools with minimap2 (Li 2018) as is used in workflows such as MAESTRO (Wang *et al.* 2020)].

The second approach, Cell Ranger ATAC by 10x Genomics, provides end-to-end analysis, from read mapping through downstream tasks like cell clustering using called peaks and counts. It is substantially faster than the traditional pipelines due to optimized components that minimize redundant computation and file I/O. However, there is still much potential for further improvement. Additionally, Cell Ranger ATAC is released under a non-free license, which permits any use or modification with only 10x Genomics’ own proprietary and patented technologies. The third, and the fastest method is Chromap (Zhang *et al.* 2021), which uses a minimizer-based (Roberts *et al.* 2004) index to map reads by generating candidate anchors from minimizer hits and aligning candidate positions to the reference. It further speeds up mapping by caching the frequently occurring reads

Some approaches, like scATAK and snATAK (Booeshaghi *et al.* 2023), use lightweight mapping methods such as pseudoalignment to single-cell and single-nucleus ATAC-seq data. However, they still rely on traditional read alignment tools to align reads to the genome to produce the accurate genomic mappings necessary to extract peaks, whose sequences are treated as experiment-specific targets for pseudoalignment (Bray *et al.* 2016). While lightweight mapping approaches have proven very successful in providing fast read mapping in transcriptomics (Bray *et al.* 2016, Patro *et al.* 2017) and metagenomics (Schaeffer *et al.* 2017, Mäklin *et al.* 2021, Skoufos *et al.* 2022, Alanko *et al.* 2023, Fan *et al.* 2024) without much loss in accuracy compared to alignment-based approaches, no method has yet used lightweight mapping directly to the genome in the context of single-cell ATAC-seq.

One may expect that such approaches might also help in improving the read mapping efficiency for genomic mapping as well. However, the large reference sequences, frequently consisting of repetitive regions in the genome, make it difficult to applying pseudoalignment. A *k-*mer can map to many locations within the same reference, which may lead to an incorrect determination of the mapping position, even when the specific reference (e.g. chromosome of origin) is determined correctly. These issues can lead both to reporting spurious mapping positions as well as missing the most likely approximate mapping location of a read, and can also lead to exacerbated multimapping effects.

Recently, KMCP (Shen *et al.* 2023) described an approach that uses a modified COBs (Bingmann *et al.* 2019) index to tackle the metagenomic profiling problem. The COBs data structure is an (approximate) presence/absence index based on the matching of some threshold fraction of k-mers between a query and the reference. To reduce the potential for spurious matching based off of genomically distant k-mers, and to ensure that the evidence supporting the existence and abundance of a taxon is not due to many reads assigned to only one narrow range of the underlying genome [akin to the strategy used in KrakenUniq (Breitwieser *et al.* 2018)], KMCP divides the metagenomic references into a fixed number of chunks, which themselves (rather than the genomes) become the set of distinct targets represented in the COBs index. Taking the success of such an approach as motivation, we propose a modified pseudoalignment approach for mapping reads to the genome, which partitions the references into “virtual colors” of fixed maximum extent. To determine the set of references (and their approximate positions) from which a read originates, the intersection across the virtual colors to which the *k-*mers in a read map is performed instead of taking the intersection over the original colors (i.e. references/chromosomes). This also necessitates careful handling of the boundaries between virtual colors (to ensure reads spanning virtual colors are not lost) as well as ensuring that duplicate mappings induced by virtual color overlap are properly identified with only a single position in the original reference space.

## 2 Contributions

- We present alevin-fry-atac, a novel tool designed for the efficient processing and mapping of single-cell ATAC-seq data. It extends the memory frugal piscem index, incorporating a modified pseudoalignment algorithm based upon the concept of virtual colors that is tailored for accurate genomic mapping without sacrificing efficiency.
- To further enhance the mapping speed, we optimized and improved the streaming querying procedure of SSHash (Pibiri 2022) (a key component of the piscem index) and additionally developed a caching mechanism to speed up the search of *k-*mers not amenable to streaming query optimizations. Both of these enhancements leverage insights about the structure of the compacted de Bruijn graph, which is a core component of our index.
- We have enhanced the processing functionalities of alevin-fry by incorporating a new, data-adaptive, parallelized, external-memory sorting procedure, which outputs a cell-barcode corrected, deduplicated, and coordinated-sorted BED file. The BED file contains all necessary information to be directly used with popular peak callers such as MACS2 (Zhang *et al.* 2008, Gaspar 2018) and other downstream tools for analyzing single-cell ATAC-seq data.
- We evaluated alevin-fry-atac on both simulated and experimental datasets and compared it with the other methods for mapping single-cell ATAC-seq data. We observed a high concordance between the peaks and clusters generated on the mapped fragment file produced by Cell Ranger ATAC and Chromap and those generated on the mapped fragment file produced by alevin-fry-atac. Alevin-fry-atac uses 33% less memory and is 2.8 times faster compared to Chromap when 32 threads are provided to process and map single-cell ATAC-seq data.
- We have integrated Alevin-fry-atac into the alevin-fry ecosystem and simpleaf (He and Patro 2023), a framework originally designed to simplify single-cell RNA-seq analysis using alevin-fry. This integration creates a unified, open-source framework that simplifies and streamlines multi-modal analysis.

## 3 Material and methods

### 3.1 Preliminaries

The piscem (Patro *et al.* 2025) index is a modular index that enables efficient retrieval of a set of references (colors), as well as all of the associated positions and orientations for a given *k-*mer *x*. Like the pufferfish (Almodaresi *et al.* 2018) index that preceded it, the piscem index is constructed over the compacted colored reference de Bruijn graph, permitting the same set of operations. However, the piscem index is substantially smaller than that of pufferfish. For a given set of reference sequences, piscem uses cuttlefish (Khan and Patro 2021) to efficiently construct the compacted colored reference de Bruijn graph and the associated tiling of the reference by unitigs. It then uses SSHash (Pibiri 2022) to create a *k-*mer to unitig mapping K→U, which maps each *k-*mer to a unitig, relative offset, orientation triplet ($u_{k},p_{k},o_{k}$). Piscem builds a dense inverted index to represent the unitig-to-reference tiling U→R, which allows recalling, for each unitig the reference sequences, and relative positions and orientations of the unitig’s occurrences in the original reference. The *k-*mer to unitig K→U and unitig-to-reference U→R can be combined to correctly and efficiently determine the mapping position (and orientation) of a *k-*mer in the reference, which might be essential for genome-centric mapping tasks. The piscem index can be constructed on different types of reference sequences(genome, transcriptome, metagenome, etc.), and the associated software can currently support the processing and mapping of single-cell RNA-seq and bulk RNA-seq data. With the introduction of alevin-fry-atac, single-cell ATAC-seq is now also supported. A complete manuscript describing the piscem index, the key details of the software implementation, and its applications to various indexing tasks is currently under preparation.

Pseudoalignment is a lightweight mapping procedure that aims to assess the “compatibility” between a read and a collection of references without specifically finding the coordinates of each base of the read in the reference. Several approaches and algorithms for pseudoalignment have been proposed. They vary from how and which set of *k-*mers of a read is selected for mapping to how the colors corresponding to the selected *k-*mers are aggregated to get the final color set. The color set denotes the references to which a read maps (Wood and Salzberg 2014, Alanko *et al.* 2023, Fan *et al.* 2024).

The pseudoalignment strategy that has been used in the current manuscript is the “hybrid” method described in Themisto (Alanko *et al.* 2023) or the “threshold-union” method described in Fulgor (Fan *et al.* 2024). Let $R=\{R_{1},R_{2},\ldots ,R_{N}\}$ denote the set of reference sequences on which we want to map a query sequence *Q*. Let K(Q) denote the set of *k-*mers for which a mapping K→U exists in the index. Given the threshold parameter τ∈(0,1], the output of pseudoalignment for a read is a subset $R_{C}\subset R$ and the relative read mapping position within a reference, such that each reference in the output $R_{C}$ appears as a mapped hit (i.e. *k-*mer match) across at least τ*|K(Q)|  *k-*mers. The mapping position of the leftmost *k-*mer hit of *Q* to the reference is assigned as the mapping position of the read to the reference.

### 3.2 Mapping and pseudoalignment using virtual colors

**Figure 1.**
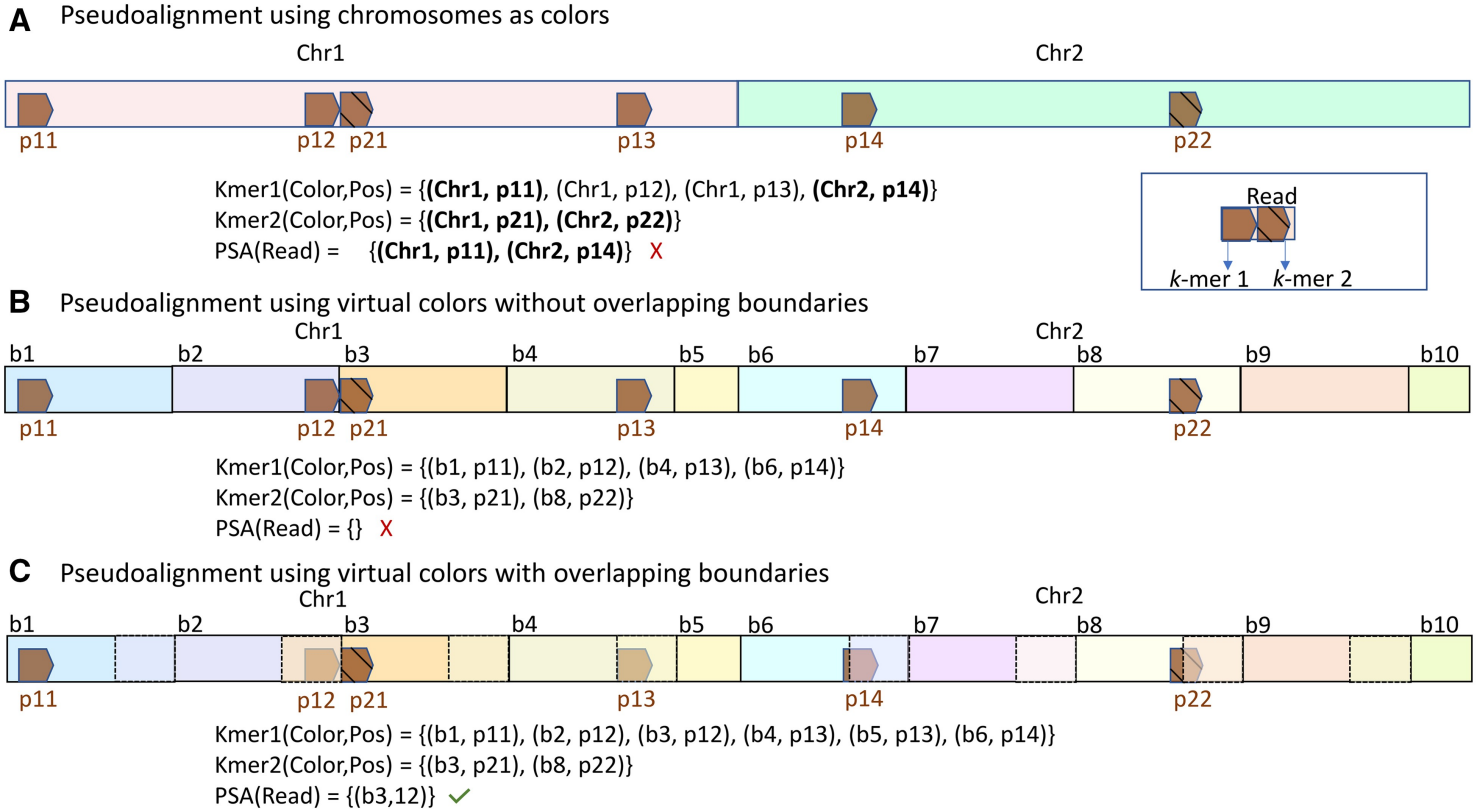
Toy example demonstrating how traditional pseudoalignment on genomic references could lead to spurious mapping or completely missing the correct mapping. In the example, we have two *k-*mers, which map to multiple references, with *k-*mer1 mapping to multiple locations within chromosome 1. The individual mapped positions within a reference are denoted by p*ij*, with *i* denoting the *k-*mer id and *j* denoting the order of the positions globally for that *k-*mer. PSA(Read) denotes the reference ids and the relative positions on the reference along which Read maps using pseudoalignment. The true mapping for the read in the example is (Chr1, p12). (A) Using traditional pseudoalignment, both Chr1 and Chr2 are selected as references from which the read originates. The position to which a read maps using pseudoalignment is governed by the offset of the left-most *k-*mer in the read for which a mapping exists in the reference. This is motivated by Kallisto and Salmon, which use a similar strategy to resolve such multimapping. If a *k-*mer maps to multiple positions within a reference, then by design, the position with the smallest coordinate is picked, which for Chr1 is p11. We thus have two false positive mappings (Chr1, p11), (Chr2, p14) and a false negative since (Chr1, p11) is not reported. (B) When the references are projected onto a set of virtual colors whose boundary spaces do not overlap, we miss the mapping if the read spans across the boundaries (b2, b3), creating an ∅ set when taking the intersection across the colors. (C) When the boundaries of virtual colors overlap, each *k-*mer is assigned two colors if it falls within the overlapping region. This ensures that the read gets mapped after pseudoalignment.

The accuracy of compatibility information generated by pseudoalignment may be similar to alignment-based approaches when the reference sequences are comparably short, which is the case for transcriptomics and metagenomics [and when the sequenced sample is devoid of non-indexed references that are sequence-similar to the indexed references (Srivastava *et al.* 2020)]. However, when directly applying pseudoalignment for mapping to a genome, where the reference sequences are large, comprising of chromosomes that often contain repetitive sequences, we observe a marked drop-off in mapping accuracy using various traditional pseudoalignment approaches. This can happen due to multiple matching locations for a *k-*mer within a single reference that can lead to false positive and false negative mapping positions during read mapping, as demonstrated in Fig. 1A.

*Construction of the virtual colors—*We propose modifying the pseudoalignment approach by projecting the original reference sequences (i.e. the original colors) onto a set of “virtual colors,” that themselves do not exist as individual elements in the index, but which will represent the targets to which queries can map. The virtual colors, conceptually, consist of a set of overlapping bins, each of fixed maximal extent, on the reference sequences. This overlap is needed since the matching *k-*mers of a read can span the boundary of multiple disjoint virtual colors (and therefore become lost) (Fig. 1B and C). The virtual colors are designed not to extend over the individual reference sequences. Thus a virtual color $b_{i}$ belonging to reference $R_{i}$ will not overlap with any virtual color $b_{j}$ belonging to reference *j*, ∀i≠j. The construction of the virtual colors is controlled by two parameters, namely $\ell_{\text{vcol}}$ and *ov_length*. The parameter *ov_length* controls how many bases a virtual color will overlap with the virtual color on its left. It should be at least equal to the read length to ensure that the entire read can fit within it. The parameter $\ell_{\text{vcol}}$ controls the total number of bases spanned by a virtual color, with most virtual colors on a reference covering $\left(ov\_\mathrm{length}+\ell_{\text{vcol}}\right)$ bases. The leftmost virtual color on a reference will cover $\ell_{\text{vcol}}$ bases. The rightmost virtual color on the reference will cover ov_length+rl bases such that $\left(B[R_{i}]-1)\cdot \ell_{\text{vcol}}+rl=L[R_{i}\right]$, where $B[R_{i}]$ denotes the total number of virtual colors contained within the reference sequence $R_{i}$ and $L[R_{i}]$ denotes the length of the reference sequence. $B[R_{i}]$ can be computed as $\mathrm{ceil}\left(\frac{L[R_{i}]}{\ell_{\text{vcol}}}\right)$. The virtual colors are created dynamically, independent of the index, not creating any overhead.

*Assigning virtual color to a k-mer—*Before starting the mapping process, we create an array *CB* of length |R|, such that:


$$CB[R_{i}]=\{\begin{array}{cc} 0, & \text{if}\;R_{i}=0\\ CB[R_{i}-1]+B[R_{i}-1] & \text{otherwise} \end{array}$$

where $CB[R_{i}]$ stores the cumulative number of virtual colors up to reference $R_{i}$. To assign a virtual color to a *k-*mer *x* for which there exists a mapping in the index, we extract the reference id, $R_{i}$, and $p_{ij}^{x}$—the relative position of the *j*-th occurrence of the *k-*mer *x* within reference *i* in the index. Corresponding to each occurrence of *k-*mer *x* on reference $R_{i}$, the color id of *k-*mer *x* is computed as $V(x)=CB[R_{i}-1]+\frac{p_{ij}^{x}}{\ell_{\text{vcol}}}$. Further, we check if the *k-*mer *x* lies in the overlapping region between the bins. If that is the case, then it is assigned an additional color id V(x)+1. This ensures that we do not miss a potential mapping of a read if it entirely (or partly) lies within the overlapping regions.

*Obtaining the final color set—*Since a *k-*mer can be assigned two virtual colors corresponding to the same matching position, it might happen that a read after pseudoalignment also gets assigned two virtual colors for that same reference position. This is not a problem for mapping single-end reads, as removing mappings with duplicate color ids and positions is straightforward. However, individual mate mappings must be merged for paired-end reads to get the final set of mapped references and the corresponding start positions. To do this, we first remove duplicate mappings for the individual mates. Two mappings *a*, *b* are deemed duplicate ↔: (ori(a)=ori(b)), (references(a)=references(b)), and (pos(p)=pos(b)), where ori(·),reference(·), and pos(·) denote the orientation, reference id (original color) and position for a mapped hit, respectively.

By design, a mapped hit will always have *at most one other duplicate* under the above criteria. These hits are deduplicated by keeping the hit with the smaller virtual color id. After removing the duplicate mapping hits for each mate, hits *a* and *b* are merged, with *a* being the mapping for mate 1 and *b* being the mapping for mate 2, if they meet the following criteria:


$$\begin{array}{c} \text{references}\left(a\right)=\text{references}\left(b\right)\\ |\text{vid}\left(a\right)-\text{vid}\left(b\right)|\leq 1\\ \text{ori}\left(a\right)\neq \text{ori}\left(b\right)\\ -{\text{dovetail}}_{\text{max}}\leq \text{pos}\left(a\right)-\text{pos}\left(b\right)\leq {\text{ins}}_{\text{max}},\text{if}\;\text{ori}\left(a\right)=r\\ -{\text{dovetail}}_{\text{max}}\leq \text{pos}\left(b\right)-\text{pos}\left(a\right)\leq {\text{ins}}_{\text{max}},\text{if}\;\text{ori}\left(a\right)=f \end{array}$$

Here vid(·) represents the virtual color id and orientation *f*, *r* represents the forward and reverse orientation, respectively. The use of values ${\text{dovetail}}_{\text{max}}=20$ and ${\text{ins}}_{\text{max}}=1000$ in this work is motivated from alevin-fry (He *et al.* 2023).

The mapped hits supported by different numbers of *k-*mers can arise when the threshold τ<1. In such cases, we want to pick the hits for which we have the highest confidence, provided by the number of *k-*mer hits. Thus, we keep track of all valid mappings for the read pair, and let $\tau^{\prime }$ denote the largest fraction of *k-*mer hits supporting any reported mapping. We subsequently filter out all merged mappings having $<\tau^{\prime }$ fraction of valid mapped *k-*mers.

To map the paired-end reads, we first try to merge the individual reads and create a unified fragment. If we are successfully able to merge the reads, then the merged fragment is mapped to the genome instead of the individual reads. We have described this in detail in *Mapping and Pseudoalignment* subsection under Methods, available as supplementary data at Bioinformatics online.

### 3.3 Speeding up *k-*mer query

A key component of efficiently mapping reads using pseudoalignment is the ability to efficiently retrieve the unitig (and relative offset and orientation within the unitig) to which the *k-*mers in a read map. While the current piscem index enables fast extraction of this information, we further optimize the querying procedure to enable even faster retrieval of the unitig and reference information associated with *k-*mers.

#### 3.3.1 Streaming queries

Consecutive *k-*mers of read are likely to map to consecutive positions on the reference(s). Consequently, many sequences of adjacent *k-*mers may also originate from the same unitig. Consider, e.g. the error (and variation) free case. If the first *k-*mer of a read that is truly drawn from the genome maps to some position *p* in unitig *u*, then the subsequent $\ell_{u}-p$  *k-*mers will map to the following positions in the same unitig, with $\ell_{u}$ denoting the distance from the end of the unitig *u*. When a *k-*mer is verified to exist in a unitig, we need not search for it anywhere else in the index, since a *k-*mer may appear at most once in the de Bruijn graph. Hence, by optimistically checking for subsequent query *k-*mers that may reside in the same unitig (which, itself, is likely in CPU cache), we can often avoid having to refer to the index entirely until the unitig of origin shifts.

Specifically, when querying a *k-*mer, we maintain a state variable. This variable stores the information about the unitig to which the previously mapped *k-*mer of the read matched, including its id, orientation, relative offset, and distance from the unitig’s end. The state variable is empty in two cases: either the first *k-*mer of a read is being queried, or the distance of the previous *k-*mer from the end of the unitig is 0 (i.e. the last *k-*mer on the unitig w.r.t the orientation of mapping).

In both these cases, the current *k-*mer is directly queried against the index. Otherwise, the next nucleotide is extracted from the unitig using the previous *k-*mer’s offset and orientation. This nucleotide is checked against the last nucleotide of the current *k-*mer. If they match, then we have the relevant mapping information for this *k-*mer. Otherwise, a query to the index is made. The mapping process becomes faster when fewer queries are made to the main index, which is made possible due to consecutive *k-*mers in the read. The approach is similar to the streaming iterator in SSHash (Pibiri 2022). However, the implementation in SSHash tracks the minimizer of the current *k-*mer and queries the corresponding minimizer buckets of the index whenever the minimizer changes, regardless of whether or not a straightforward extension would succeed. In our implementation, by exploiting the structure of the unitigs and the guarantees provided by the de Bruijn graph, we avoid even this lookup, which we find can lead to a substantial (30%–40%) improvement in average lookup speed when querying each *k-*mer in a read.

#### 3.3.2 Caching terminal *k*-mers

We aim to exploit the observation that chromatin profile enrichment is confined to specific genomic regions. The mapping information for *k-*mers can be efficiently extracted if they lie within the same unitig. However, a query to the index is always necessary when *k-*mer resides on a different unitig. We thus implement a caching mechanism to enable efficient retrieval of the mapping information of the terminal *k-*mers (first or last on a unitig). Specifically, we suspect that mapped reads will repeatedly traverse specific unitig paths in the underlying de Bruijn graph, and so that by inserting the terminal *k-*mers in these unitigs in a small and fast cache, we can often avoid lookups in the main index even when moving between unitigs.

The cache is implemented as a shared, concurrent, hash map (we use the Boost unordered_concurrent_flat_map) maintained across all mapping threads. Hash map keys are terminal *k-*mers, and the values are the results of querying the index for these *k-*mers. The map has a fixed size (default 5, 000, 000 entries) and follows a write-once policy, meaning, that once a key-value pair is added, it is not replaced.

For each *k-*mer, we first determine if it is terminal, after which the cache is checked. If the *k-*mer is present in the cache, the corresponding unitig information is retrieved directly. Otherwise, a full query is performed on the index, and if the *k-*mer is found and the map size permits, the *k-*mer is added to the cache.

To identify terminal *k-*mers, we leverage the state information. Specifically, a *k-*mer is labeled as terminal if the distance of the previous *k-*mer from the unitig’s end is less than the distance between the offsets of the previous and the current *k-*mers. This policy allows us to avoid even attempting to consult the cache when we expect the next *k-*mer to occur within the same unitig, and to only consult and modify the cache for the terminal *k-*mers.

We note that while the idea of using a cache to speed up mapping to a data-dependent subset of the genome is inspired by Chromap; the nature of our cache, the elements that are cached and how it is implemented are completely different from that proposed in Chromap.

### 3.4 Alevin-fry-atac pipeline

We now describe the alevin-fry-atac pipeline for preprocessing single-cell ATAC-seq data. The first step is to map all the reads using the modified pseudoalignment algorithm described above. The default output of this phase is a RAD (reduced alignment data) format file, which is a chunk-based, binary file optimized for machine parsing that encodes the relevant information necessary for subsequent data processing (He *et al.* 2022). It is worth noting that the single-cell ATAC-seq variant of such files carries some distinct information from the single-cell RNA-seq variant (e.g. it includes positions for each mapping, but does not include UMI information).

There exist two more steps in the pipeline: barcode correction, followed by sorting and deduplication. During barcode correction, we try to correct the barcodes of the mapped fragments, not present in the permit list file provided by 10x Genomics, to their nearest neighbor present in that file. The fragment barcodes of a sample will be a subset of the permit list. After the barcodes are corrected, the mapped fragments are sorted by their position, and all duplicates of a mapped fragment are removed to produce a BED file as output. The BED file contains the reference name, start position, end position, cell barcode and the total number of duplicates (including the fragment itself). We describe these steps in more detail in the Methods section, available as supplementary data at Bioinformatics online.

### 3.5 Dataset and the analysis pipeline

We evaluated and compared the performance of the different methods on three simulated and three experimental datasets. Mason (Holtgrewe 2010) was used to generate the simulations for reads of length 50, 100, 150 bases. One million paired-end fragments for each read length were created with a probability mismatch 0.25, according to the Illumina model in the simulator. The experimental datasets used in this study include the Human 10K PBMC, Mouse 8K Cortex and Human 3K Brain, respectively, some of which have been utilized for analysis and benchmarking in the previous studies. The datasets have been downloaded from 10x Genomics website, for which the URLs are provided in Table 2, available as supplementary data at Bioinformatics online. The Human 10K PBMC and Mouse 8K Cortex datasets are single-cell ATAC-seq datasets obtained using Chromium Controller, v2 chemistry. The Human 3K Brain dataset is a multiomic dataset obtained using Epi Multiome ATAC + Gene Expression, v1.0 chemistry. In this paper, we have utilized the single-cell ATAC-seq modality for this dataset. Chromap was run with “–preset atac” argument when the analysis involved both the mapping and processing of single-cell ATAC-seq data. It was run without any “–preset” argument, when the goal of the analysis was to map reads to the genome. Details regarding the datasets, analysis pipeline, experimental setup, evaluation, benchmarking, and the software that have been used are provided in detail in *Analysis pipeline* subsection under Methods, available as supplementary data at Bioinformatics online.

**Table 1.** Mapping accuracy on the simulated data for the different methods.

Method	Read length		
	50	100	150
Alevin-fry-atac (k=25,τ=0.7,$\ell_{\text{vcol}}=1,000$)	93.72	96.05	96.68
Chromap (w = 7, k = 17)	95.48	97.11	97.61
Bowtie2	95.71	97.16	97.65

**Table 2.** Percentage overlap between the peaks pairwise for the different methods across the datasets.^a^

Method/dataset	Alevin-fry-atac versus Chromap	Alevin-fry-atac versus Cell Ranger ATAC	Chromap versus Cell Ranger ATAC
Human 10K PBMC	96.13 (97.03)	96.27 (97.01)	98.67 (97.49)
Mouse 8K Cortex	97.62 (97.21)	96.79 (93.07)	97.49 (94.13)
Human 3K Brain	92.72 (95.55)	N/A	N/A

a Depending on which method is used as a denominator, the percentage overlap can change. The values in the parentheses denote the overlap when the second method was used as the denominator. N/A entries denote that Cell Ranger ATAC was not run on that dataset.

## 4 Results

### 4.1 Mapping accuracy on the simulated data

*Evaluating the effect of different parameters for alevin-fry-atac—*As described in the methods section, alevin-fry-atac includes several parameters: the *k-*mer size *k*, pseudoalignment threshold τ and $\ell_{\text{vcol}}$, which controls the number of bases a virtual color will span (with most virtual colors spanning $\ell_{\text{vcol}}+ov\_\mathrm{length}$ bases). The mapping accuracy is closely tied to the value of these parameters. To evaluate this, we generated simulated datasets with varying read lengths (50, 100, 150 base pairs) and on each dataset, tested the accuracy under different combinations of these parameters. For each evaluation, two parameters were held constant while the third was varied. The following parameter values were tested:


$$\begin{array}{c} \ell_{\text{vcol}}=\{1000,10000,100000,1000000,\mathrm{chr}\}\\ k=\{23,25,27,29\},\\ \tau =\{0.6,0.7,0.8,1\}. \end{array}$$

*chr* implies mapping is done using chromosome sequence as colors.

**Figure 2.**
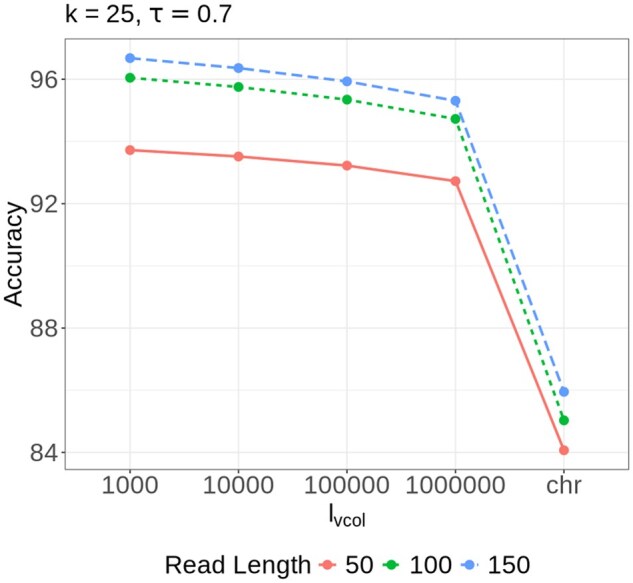
Evaluating accuracy across different read lengths for alevin-fry-atac by varying $\ell_{\text{vcol}}$ for k=25,τ=0.7. The *x*-axis label *chr* implies mapping is done using chromosome sequence as colors (removing the impact of virtual colors on mapping procedure).

**Figure 3.**
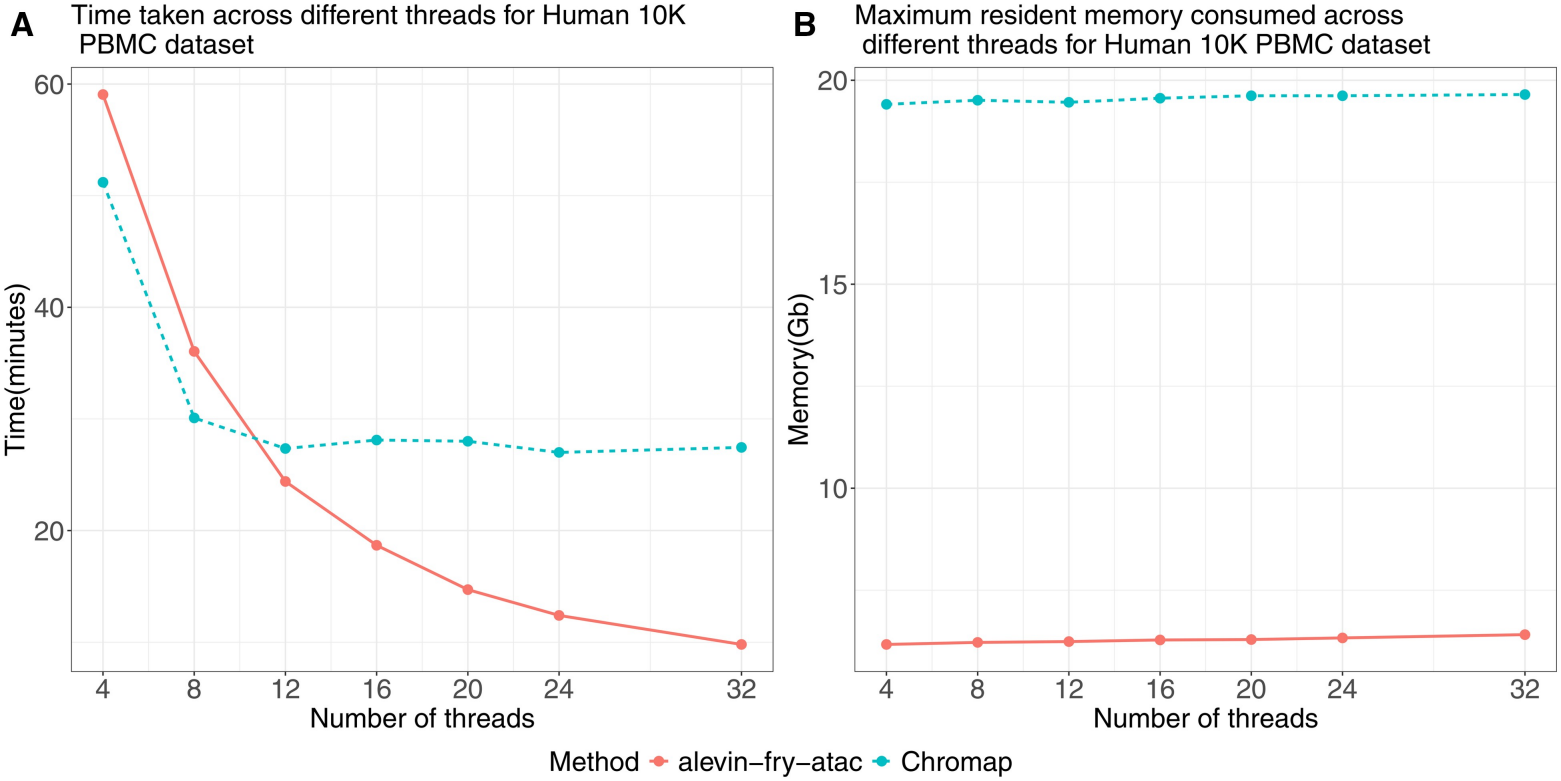
Comparing the time taken (A) and maximum resident memory (B) consumed by alevin-fry-atac and Chromap for the Human 10K PBMC dataset across the different threads.

The mapping accuracy for each parameter combination is detailed in Results, available as supplementary data at Bioinformatics online and visualized in Figs 1–12, available as supplementary data at *Bioinformatics* online. In summary, we observe that virtual colors, regardless of the $\ell_{\text{vcol}}$ value, *substantially* enhance the mapping accuracy compared to using chromosomes as colors for pseudoalignment. It has the largest impact on accuracy of all the parameters, where the impact is consistent across different read lengths and parameter combinations. Specifically, the accuracy drops from 93.7% to 84% when the $\ell_{\text{vcol}}$ value is changed from 1000 to *chr*, with k=25,τ=0.7 for reads of length 50 (Fig. 2).

For reads of length 50, the highest accuracy is observed at k=25, while for reads of length 100 and 150, accuracy improves at larger *k* values. Additionally, accuracy generally decreases as τ increases from 0.6 to 1.

*Comparing the mapping accuracy with the other methods—*We next compare the mapping accuracy of alevin-fry-atac with Bowtie2 (Langmead and Salzberg 2012) (which does full read alignment) and Chromap on the simulated dataset which is summarized in Table 1. Similar to alevin-fry-atac, the other methods also demonstrate higher accuracy for longer read lengths. Among the methods, Bowtie2 has the highest accuracy, followed closely by Chromap, while alevin-fry shows slightly lower accuracy across all the evaluated read lengths. Specifically, for reads of length 50, the mapping accuracy of alevin-fry is ∼2% lower than Bowtie2 and around ∼1% lower for reads of length 150.

### 4.2 Experimental datasets

*Mapping Rate—*We also looked at the impact of varying the different parameters of alevin-fry-atac on the overall mapping rate for the different experimental datasets, namely Human 10K PBMC, Mouse 8K Cortex and Human 3K Brain (Fig. 13, available as supplementary data at *Bioinformatics* online). As with the simulated dataset, we also find a decrease in mapping rate as $\ell_{\text{vcol}}$ increases for different *k* and τ values, with the fall being the sharpest when chromosomes are used as colors (i.e. when virtual colors are disabled). Further, since we have relatively shorter read lengths in these datasets, a higher mapping rate is observed at lower values of *k*. The highest mapping rate is observed for k=25, followed closely by k=23 for the human datasets, while for the mouse dataset the highest mapping rate is achieved at k=23 followed by k=25, with the lowest values observed at k=31 across all datasets. We observe an overall higher mapping rate for alevin-fry-atac using $k=25,\tau =0.7,\ell_{\text{vcol}}=1,000$  (98.27%,97.73% and 96.99) for the Human 10K PBMC, Mouse 8K Cortex and Human 3K Brain datasets respectively while for Chromap (without using the—preset atac argument) is (95.31%,95.56% and 94.50%). The high mapping accuracy observed for our proposed pseudoalignment approach for genomic mapping, which is comparable to alignment-based approaches, makes us believe it can also be used for mapping reads obtained from other genomic modalities such as Chip-seq, Hi-C, etc., in addition to (sc)ATAC-seq, which we explore further in the section below.

*Comparing peaks—*After obtaining the mapped fragment file—which has been barcode-corrected, deduplicated, and coordinate-sorted—a key step in single-cell ATAC-seq analysis is peak identification. Peaks represent genomic regions with significant enrichment of sequencing reads. To identify peaks, we used MACS2 on the mapped fragment files generated by alevin-fry-atac, Chromap and Cell Ranger ATAC across different datasets. The total number of bases covered by the peaks is provided in Table 3, available as supplementary data at Bioinformatics online, which also includes the bases covered by peaks identified using the custom Cell Ranger ATAC peak caller, which could only be run on the Cell Ranger ATAC fragment file. Additionally, we did not process the Human 3K Brain dataset with Cell Ranger ATAC, as it is a multi-omic dataset and required processing with a different software, namely Cell Ranger Arc.

**Table 3.** Evaluating the concordance between the clusters obtained for the different methods pairwise across the datasets.^a^

Method/dataset	Alevin-fry-atac versus Chromap	Alevin-fry-atac versus Cell Ranger ATAC	Chromap versus Cell Ranger ATAC
Human 10K PBMC	0.87, 0.91	0.93, 0.94	0.87, 0.91
Mouse 8K Cortex	0.95, 0.96	0.94, 0.94	0.93, 0.94
Human 3K Brain	0.89, 0.91	N/A	N/A

a The two entries per cell, separated by comma, denote Adjusted Rand Index (ARI) and Normalized Mutual Information (NMI). N/A entries denote that Cell Ranger ATAC was not run on that dataset.

We observed that the peaks produced by the Cell Ranger ATAC output cover the largest number of bases across all datasets. Notably, peaks identified using Cell Ranger ATAC custom peak caller cover more bases than those identified by MACS2 (Gaspar 2018). We next analyzed the impact of using different processing methods while using the same peak caller (MACS2). We observed that peaks produced by alevin-fry-atac cover more bases compared to Chromap on the human datasets, while on mouse datasets, Chromap covered more bases. The peak overlap between the methods exceeds 90% across all datasets, and in most cases, it is greater than 95% (Table 2). The overlap between Chromap and Cell Ranger ATAC is slightly higher than the overlap between alevin-fry-atac and Cell Ranger ATAC. We also extracted the unique peak regions for Chromap and alevin-fry-atac individually by doing a pairwise comparison between them, and looked at their overlap with the bases covered by soft-masked regions and the blacklist regions which is provided in Table 4, available as supplementary data at Bioinformatics online. 55% and 47% of the unique peaks obtained for Chromap and alevin-fry-atac map to the soft-mask regions, whereas only 1.1% and 1.4% of them map to the blacklist regions.

**Table 4.** Time and maximum resident memory consumed by the different methods for mapping scATAC-seq reads.^a^

Method	Memory (GB)	Time (min)
Human 10K PBMC		
Cell Ranger ATAC^b^	14.9	222
Alevin-fry-atac	6.4	9.80
Chromap	19.7	27.45
Mouse 8K Cortex		
Cell Ranger ATAC^b^	8.6	190
Alevin-fry-atac	5.62	7.91
Chromap	18.8	21.85

a For both Chromap and alevin-fry-atac results are reported using 32 threads.

b For Cell Ranger ATAC, it is not possible to run and thus benchmark each step individually, thus the time and memory consumption also include other tasks such as peak calling and clustering.

*Comparing the clusters—*We clustered the cells using Signac (Stuart *et al.* 2021) for the different methods across the different datasets. We performed a pairwise comparative analysis between the clusters obtained for different methods. Across datasets, we observed a large overlap between clusters (Figs 14 and 15, available as supplementary data at *Bioinformatics* online). On the Human 10K PBMC dataset, there was less crossover (i.e. more concordance) between the clusters obtained for Cell Ranger ATAC and alevin-fry-atac compared to other pairwise comparisons. Similarly, the UMAP plots (Figs 16–18, available as supplementary data at *Bioinformatics* online) revealed very similar cluster projections.

Quantitatively, we evaluated the cluster labels (Table 3) using Adjusted Rand Index (ARI) and Normalized Mutual Information (NMI). High concordance was observed across all datasets. on the Human 10K PBMC dataset, ARI and NMI for the comparison between alevin-fry-atac and Cell Ranger ATAC were higher than the other pairwise comparisons. On the Mouse 8K Cortex dataset, ARI and NMI were highest for comparison between alevin-fry-atac and Chromap. Similarly, for Human 3K Brain dataset. NMI >0.9 is observed between alevin-fry-atac and Chromap. Although all methods showed high concordance with close values, clusters produced using alevin-fry-atac peaks consistently demonstrated equal or higher concordance with clusters from Cell Ranger ATAC compared to those from Chromap.

### 4.3 Memory and running time

Finally, we benchmarked the memory usage and runtime of alevin-fry-atac and Chromap for mapping and processing by evaluating their performance on Human 10K PBMC and Mouse 8K Cortex datasets using varying numbers of threads (Fig. 3, Fig. 19, available as supplementary data at *Bioinformatics* online). Across both datasets, Chromap was faster than alevin-fry-atac when using 4 or 8 threads. However, starting at 12 threads, alevin-fry-atac began to outperform Chromap, becoming substantially faster at 32 threads, taking only 9.80 and 7.91 minutes for the Human 10K PBMC and Mouse 8K Cortex datasets respectively, where Chromap takes 27.45 and 21.85 minutes. Notably, Chromap showed poor scalability beyond 12 threads, whereas alevin-fry-atac demonstrated significantly improved scalability.

In terms of memory usage, alevin-fry-atac consistently consumed substantially less memory than Chromap. Alevin-fry-atac used approximately 6.4 and 5.6 Gbs for the Human 10K PBMC and Mouse 8K Cortex datasets (Fig. 3B, Fig. 19B, available as supplementary data at *Bioinformatics* online), compared to 19.7, 18.8 Gbs used by Chromap. Additionally, Chromap’s mapping-only mode (which only does read mapping and no other processing) and referred to as (Chromap (Map Only)) used 39.4, 35 Gbs for the two datassets, respectively (Figs 20 and 21, available as supplementary data at Bioinformatics online). The benchmarking results are summarized in Table 4 for 32 threads, which also includes the results for Cell Ranger ATAC. However, it is important to note that the metrics reported for Cell Ranger ATAC also include the time spent on peak calling and other downstream analyses, as the tool does not allow one to execute each step individually and thus its reported metrics are not directly comparable with the other methods.

## 5 Discussion

We have introduced alevin-fry-atac, which provides a computationally efficient, memory-frugal, scalable and accurate framework for processing and mapping single-cell ATAC-seq data. It uses only 33% of the memory required by Chromap and less than half of the memory used by Cell Ranger ATAC when processing single-cell ATAC-seq data (and alevin-fry-atac uses only 17% of the memory required by Chromap when the latter is not provided with a permit list during mapping). Since Chromap only maps the reads corresponding to the barcodes present in the permit list file, this could be one reason why we observe an increased time usage in the mapping-only mode. Internally, alevin-fry-atac uses the piscem index, which is small and versatile and enables mapping reads from other technologies such as bulk RNA-seq, single-cell RNA-seq and now single-cell ATAC-seq.

The enhanced streaming query and the introduction of cache help in significantly improving the speed and scalability of alevin-fry-atac. When using 12 threads, alevin-fry-atac outperforms Chromap in terms of speed and achieves even greater gains at the higher thread counts. At 32 threads, alevin-fry-atac processes, and maps single-cell ATAC-seq data 2.8 times faster than Chromap. The introduction of virtual colors, and modifications to the pseudoalignment procedure enable accurate read mapping, achieving precision close to alignment-based methods. This level of accuracy would not have been attainable without using virtual colors. Despite the slightly lower mapping accuracy compared to alignment-based approaches, this does not seem to impact downstream analyses. Across experimental datasets, we observe a very high concordance between the peaks and clusters produced by alevin-fry-atac and those produced by the other tested methods. The alevin-fry and simpleaf ecosystem already supports the processing of single-cell RNA-seq. The added support for single-cell ATAC-seq makes it the only fully open-source software that directly supports both modalities, simplifying and facilitating multi-modal analysis. We thus believe that alevin-fry-atac is a viable and strong alternative to the other methods, combining efficiency, scalability, and accuracy in single-cell ATAC-seq data analysis.

While the main focus in this manuscript has been on mapping single-cell ATAC-seq data, it should be trivial to extend and apply pseudoalignment using virtual colors to other genome-centred mapping-based tasks and modalities, such as DNase-seq, Chip-seq and Hi-C.

Finally, for our pseudoalignment strategy, we have queried all *k-*mers and utilized the “threshold-union” approach. However, other lightweight mapping strategies such as selective alignment (Srivastava *et al.* 2020) and pseudoalignment with structural constraints (He *et al.* 2022) can also be explored to see how they impact speed and mapping accuracy. Finally, all pipelines, including alevin-fry-atac, currently discard reads that map to more than one location. Such reads constitute 4−10% of the total reads in the datasets analyzed in this manuscript and might have a non-trivial impact on downstream analysis (Morrissey *et al.* 2024). One interesting future direction would be to develop methods to resolve the origin of such reads probabilistically using methods such as expectation-maximization.

## Supplementary Material

btaf234_Supplementary_Data

## Data Availability

The alevin-fry-atac software is written in Rust and C++ and is freely available under BSD 3-Clause license, as a free and open-source tool at https://github.com/COMBINE-lab/alevin-fry-atac. The scripts that have been used to generate the results in this manuscript are provided at https://github.com/NPSDC/alevin-fry-atac-paper-scripts.

## References

[btaf234-B1] Alanko JN , Vuohtoniemi J, Mäklin T et al Themisto: a scalable colored k-mer index for sensitive pseudoalignment against hundreds of thousands of bacterial genomes. Bioinformatics 39:i260–9, 2023. 37387143 10.1093/bioinformatics/btad233 PMC10311346

[btaf234-B2] Almodaresi F , Sarkar H, Srivastava A et al A space and time-efficient index for the compacted colored de Bruijn graph. Bioinformatics 2018;34:i169–77. 29949982 10.1093/bioinformatics/bty292 PMC6022659

[btaf234-B3] Bingmann T , Bradley P, Gauger F et al COBS: A Compact Bit-Sliced Signature Index. Berlin, Heidelberg: Springer International Publishing, 2019, 285–303. 10.1007/978-3-030-32686-9_21

[btaf234-B4] Booeshaghi AS , Gao F, Pachter L. Assessing the multimodal tradeoff. bioRxiv, https://www.biorxiv.org/content/early/2023/04/18/2021.12.08.471788, 2023, preprint: not peer reviewed.

[btaf234-B5] Bray NL , Pimentel H, Melsted P et al Near-optimal probabilistic RNA-seq quantification. Nat Biotechnol 2016;34:525–7. 27043002 10.1038/nbt.3519

[btaf234-B6] Breitwieser FP , Baker DN, Salzberg SL. KrakenUniq: confident and fast metagenomics classification using unique k-mer counts. Genome Biol 2018;19:198. 10.1186/s13059-018-1568-0 30445993 PMC6238331

[btaf234-B7] Buenrostro JD , Giresi PG, Zaba LC et al Transposition of native chromatin for fast and sensitive epigenomic profiling of open chromatin, DNA-binding proteins and nucleosome position. Nat Methods 2013;10:1213–8. 24097267 10.1038/nmeth.2688 PMC3959825

[btaf234-B8] Buenrostro JD , Wu B, Litzenburger UM et al Single-cell chromatin accessibility reveals principles of regulatory variation. Nature 2015;523:486–90. 26083756 10.1038/nature14590 PMC4685948

[btaf234-B9] Cusanovich DA , Daza R, Adey A et al Multiplex single-cell profiling of chromatin accessibility by combinatorial cellular indexing. Science 2015;348:910–4. 25953818 10.1126/science.aab1601 PMC4836442

[btaf234-B11] Fan J , Khan J, Singh NP et al Fulgor: a fast and compact k-mer index for large-scale matching and color queries. Algorithms Mol Biol 2024;19:3. 38254124 10.1186/s13015-024-00251-9 PMC10810250

[btaf234-B12] Gaspar JM. Improved peak-calling with MACS2. bioRxiv, https://www.biorxiv.org/content/early/2018/12/17/496521, 2018, preprint: not peer reviewed.

[btaf234-B13] He D , Patro R. simpleaf: a simple, flexible, and scalable framework for single-cell data processing using alevin-fry. Bioinformatics 2023;39:btad614. 37802884 10.1093/bioinformatics/btad614 PMC10580267

[btaf234-B14] He D , Soneson C, Patro R. Understanding and evaluating ambiguity in single-cell and single-nucleus RNA-sequencing. bioRxiv, 10.1101/2023.01.04.522742, 2023, preprint: not peer reviewed.

[btaf234-B15] He D , Zakeri M, Sarkar H et al Alevin-fry unlocks rapid, accurate and memory-frugal quantification of single-cell RNA-seq data. Nat Methods 2022;19:316–22. 35277707 10.1038/s41592-022-01408-3 PMC8933848

[btaf234-B16] Holtgrewe M. Mason—A Read Simulator for Second Generation Sequencing Data. Technical Report FU Berlin, October 2010. http://publications.imp.fu-berlin.de/962/

[btaf234-B17] Khan J , Patro R. Cuttlefish: fast, parallel and low-memory compaction of de Bruijn graphs from large-scale genome collections. Bioinformatics 2021;37:i177–86. 34252958 10.1093/bioinformatics/btab309 PMC8275350

[btaf234-B18] Langmead B , Salzberg SL. Fast gapped-read alignment with Bowtie 2. Nat Methods 2012;9:357–9. 22388286 10.1038/nmeth.1923 PMC3322381

[btaf234-B19] Lara-Astiaso D , Weiner A, Lorenzo-Vivas E et al Chromatin state dynamics during blood formation. Science 2014;345:943–9. 25103404 10.1126/science.1256271 PMC4412442

[btaf234-B20] Li H. Minimap2: pairwise alignment for nucleotide sequences. Bioinformatics 2018;34:3094–100. 29750242 10.1093/bioinformatics/bty191 PMC6137996

[btaf234-B21] Li H , Durbin R. Fast and accurate short read alignment with Burrows–Wheeler transform. Bioinformatics 2009;25:1754–60. 19451168 10.1093/bioinformatics/btp324 PMC2705234

[btaf234-B22] Li H , Handsaker B, Wysoker A et al; 1000 Genome Project Data Processing Subgroup. The sequence alignment/map format and SAMtools. Bioinformatics 2009;25:2078–9. 19505943 10.1093/bioinformatics/btp352 PMC2723002

[btaf234-B23] Mäklin T , Kallonen T, David S et al High-resolution sweep metagenomics using ultrafast read mapping and inference [version 2; peer review: 2 approved]. *Wellcome Open Res* 2021;**5**:14. 10.12688/wellcomeopenres.15639.2 PMC8543175 34746439

[btaf234-B24] Morrissey A , Shi J, James DQ et al Allo: Accurate allocation of multi-mapped reads enables regulatory element analysis at repeats. *Genome Res* 2024;**34**:937–51. 10.1101/gr.278638.123 PMC11293539 38986578

[btaf234-B25] Patro R , Duggal G, Love MI et al Salmon provides fast and bias-aware quantification of transcript expression. Nat Methods 2017;14:417–9. 28263959 10.1038/nmeth.4197 PMC5600148

[btaf234-B26] Patro R , Khan J, Pibiri GE et al The piscem index. 2025. 10.5281/zenodo.15096903

[btaf234-B27] Pibiri GE. Sparse and skew hashing of k-mers. Bioinformatics 2022;38:i185–94. 35758794 10.1093/bioinformatics/btac245 PMC9235479

[btaf234-B28] Picard Toolkit. Broad Institute. 2019.

[btaf234-B29] Preissl S , Fang R, Huang H et al Single-nucleus analysis of accessible chromatin in developing mouse forebrain reveals cell-type-specific transcriptional regulation. Nat Neurosci 2018;21:432–9. 29434377 10.1038/s41593-018-0079-3 PMC5862073

[btaf234-B30] Roberts M , Hayes W, Hunt BR et al Reducing storage requirements for biological sequence comparison. Bioinformatics 2004;20:3363–9. 15256412 10.1093/bioinformatics/bth408

[btaf234-B31] Schaeffer L , Pimentel H, Bray N et al Pseudoalignment for metagenomic read assignment. Bioinformatics 2017;33:2082–8. 28334086 10.1093/bioinformatics/btx106 PMC5870846

[btaf234-B32] Schep AN , Buenrostro JD, Denny SK et al Structured nucleosome fingerprints enable high-resolution mapping of chromatin architecture within regulatory regions. Genome Res 2015;25:1757–70. 26314830 10.1101/gr.192294.115 PMC4617971

[btaf234-B33] Shen W , Xiang H, Huang T et al KMCP: accurate metagenomic profiling of both prokaryotic and viral populations by pseudo-mapping. Bioinformatics 2023;39:btac845. 36579886 10.1093/bioinformatics/btac845 PMC9828150

[btaf234-B34] Skoufos G , Almodaresi F, Zakeri M et al AGAMEMNON: an accurate metaGenomics and MEtatranscriptoMics quaNtificatiON analysis suite. Genome Biol 2022;23:39. 35101114 10.1186/s13059-022-02610-4 PMC8802518

[btaf234-B35] Srivastava A , Malik L, Sarkar H et al Alignment and mapping methodology influence transcript abundance estimation. Genome Biol 2020;21:239. 10.1186/s13059-020-02151-8 32894187 PMC7487471

[btaf234-B36] Starks RR , Biswas A, Jain A et al Combined analysis of dissimilar promoter accessibility and gene expression profiles identifies tissue-specific genes and actively repressed networks. Epigenet Chromatin 2019;12:16. 10.1186/s13072-019-0260-2 PMC6385419 30795793

[btaf234-B37] Stuart T , Srivastava A, Madad S et al Single-cell chromatin state analysis with Signac. Nat Methods 2021;18:1333–41. 34725479 10.1038/s41592-021-01282-5 PMC9255697

[btaf234-B38] Wang C , Sun D, Huang X et al Integrative analyses of single-cell transcriptome and regulome using MAESTRO. Genome Biol 2020;21:198. 32767996 10.1186/s13059-020-02116-x PMC7412809

[btaf234-B39] Wood DE , Salzberg SL, Kraken: ultrafast metagenomic sequence classification using exact alignments. Genome Biol 2014;15:1–12. 10.1186/gb-2014-15-3-r46 PMC4053813 24580807

[btaf234-B40] Zhang H , Song L, Wang X et al Fast alignment and preprocessing of chromatin profiles with Chromap. Nat Commun 2021;12:6566. 34772935 10.1038/s41467-021-26865-w PMC8589834

[btaf234-B41] Zhang Y , Liu T, Meyer CA et al Model-based analysis of ChIP-Seq (MACS). Genome Biol 2008:9:R137–9. 18798982 10.1186/gb-2008-9-9-r137 PMC2592715

