# Supplementary material for: Alevin-fry-atac enables rapid and memory frugal mapping of single-cell ATAC-seq data using virtual colors for accurate genomic pseudoalignment
Source: Bioinformatics. 2025 Jul 15;41(Suppl 1):i237–45. doi: 10.1093/bioinformatics/btaf234 (PMC12261413; doi:10.1093/bioinformatics/btaf234)
Supplement: btaf234_Supplementary_Data [file btaf234_supplementary_data.pdf]

Supplementary material

Methods

Mapping and Pseudoalignment

Merging paired-end reads before mapping — Before mapping the individual ends of a paired-end read, we try to see if we can merge the reads to create a single fragment before mapping. This idea has been suggested and adopted in several existing read-mapping tools (e.g. `STAR` (2) attempts to merge overlapping paired-end reads before mapping, and `Chromap` uses this approach for adaptor trimming (8)). We first try to merge the reads in dovetail orientation and, if that is not possible, then attempt to find an overlap merge. For a dovetail merge, the prefix of one mate should match the suffix of the reverse complement of the other mate. For an overlap merge, the suffix of one mate should intersect with the prefix of the reverse complement of the other mate. For the merge to be successful, there should be at least an overlap of a certain number of bases between the mates (we use a default of 30). If the mates can be merged, then the merged fragment and not the individual mates are mapped to the reference.

`Alevin-fry-atac` pipeline

Cell barcode correction — After mapping, the next step is cell barcode correction. While various approaches to barcode detection and correction are possible (`alevin-fry` implements several distinct modes), for `alevin-fry-atac` we currently only support correcting to an unfiltered permit list. The permit list file contains a list of experiment-independent barcodes that are a superset of the barcodes that should be observed in any given sample. We first scan through the RAD file and count the number of times a barcode corresponding to the mapped record appears (exactly). If a cell or barcode has at least `min-reads` records (default 10) mapping to it, and is also in the whitelist, it is marked as present. For all the other mapped records corresponding to cells not currently marked as present, a nearest neighbor search is performed on the barcode against the list of present cells. If a barcode has a unique (i.e. only one) present neighbor at edit distance 1, that barcode is corrected to that of the neighbor. All other records are ignored.

Sorting and Deduplication — In this step, a coordinate-sorted BED file is produced. First, a collection of temporary files is created, each corresponding to contiguous ranges of the underlying genome. The input RAD file is parsed, and all records are routed to their appropriate temporary file based on their starting mapping location. The temporary files are then individually sorted (in parallel), and the sorted files are merged (in genomic coordinate order) to produce a BED file. During the sorting process, the duplicate entries for a record (i.e., entries with the same reference, start position, end position, and barcode) are collapsed, and the corresponding number of duplicate entries is stored. For each mapped fragment, the BED file contains the reference name, start position, end position, cell barcode and the total number of duplicates (including the fragment itself). The BED file can then be passed to a peak caller such as `MACS2` 2 (3), the output of which, along with the original BED file containing the counts, forms the input for downstream analysis.

Analysis pipeline

The analysis pipelines used to carry out the experiments in this manuscript were created using `Snakemake` (4). Below, we describe how the individual experiments were carried out and evaluated.

Reference genomes — To generate the simulated datasets for analyzing the experimental datasets obtained for Homo Sapiens (humans), reference GRCh38 was used and for the datasets obtained for Mus Musculus (Mouse), reference GRCm38 was utilized, both of which were obtained from Ensembl with release version 98 (7). The URLs for the reference sequences are provided in Table S1.

Running Chromap — Depending on the underlying use-case of the analysis, `Chromap` has been run with and without the '–preset atac' argument. Chromap is designed to handle different genomic modalities, including ATAC-seq. When the '–preset' argument is provided, Chromap applies parameters for mapping specific to that modality. Additionally, it might perform other tasks for processing data from a given modality, such as barcode correction and deduplication for single-cell ATAC-seq data when using '–preset atac' mode. Specifically, in this mode, when the whitelist file is provided, `Chromap` does not necessarily map all the reads to the genome, filtering out many fragments. As a result, we cannot estimate the mapping rate over the entire read set. When evaluating the mapping performance alone, `Chromap` is run without the '–preset atac' argument. This is needed for computing the mapping accuracy or mapping rate, or to estimate the computational performance only for mapping. On the other hand, to evaluate the overall mapping and processing, the '–preset atac' argument is used when running `Chromap` on single-cell ATAC-seq data.

Simulated data — `Chromap` was run without any `--preset` arguments. For `Bowtie2`, reads were aligned by setting the maximum fragment length (`X`) to 2000. These settings were taken from `Chromap`'s experiment repository on GitHub. `Alevin-fry-atac` always maps all the records irrespective of the whitelist file. The accuracy of the mappings was evaluated using a `Python` script that was originally used in the stobemers (5) manuscript.

Experimental datasets — `Human 3K Brain` is a multiomic dataset containing both single-cell RNA-seq and single-cell ATAC-seq data. For our analysis, we only utilized the single-cell ATAC-seq data. `Cell Ranger ATAC` was run only on the `Human 10K PBMC` and `Mouse 8K Cortex` datasets, as it could not process the `Human 3K Brain` dataset. This limitation arises because `Human 3K Brain` a multiomic dataset, and even to process single-cell ATAC-seq data, it requires a different software – `Cell Ranger Arc`. We did not run this software in this manuscript, as it could not be easily compared with the other methods. The downstream pipelines for analyzing ATAC-seq data, such as `Signac` (6) require as input a `tabix`-indexed fragment file, which is typically produced in the `Cell Ranger ATAC` pipeline. Therefore, a tabix index was created for the fragment files generated by both `alevin-fry-atac` and `Chromap` for downstream analysis. Peak calling was performed using `MACS2` 2 (3). The resulting peaks and the fragment files were then passed to `Signac` for quality control and clustering.

Extracting soft-mask and blacklist regions The soft-masked GRCh38 reference genome fasta file was downloaded from Ensembl with release version 98 (7). Custom python scripts were used to extract the coordinates for the soft-masked regions in a BED file. For the blacklist regions, *blacklist_hg38_unified* object in the `Signac` `R` package was used (1).

Benchmarking — We benchmark both genomic read mapping and the end-to-end pipeline for processing and mapping single-cell ATAC-seq data for the different methods using `/usr/bin/time` command. For `alevin-fry-atac`, this process is divided into three steps: mapping reads to the genome, correcting barcodes from a whitelist file, and deduplication with sorting. Each step was benchmarked individually, and its execution times were summed to calculate the total runtime for `alevin-fry-atac`. `Chromap` was benchmarked with and without using the `--preset atac` arguments. When this argument is used, then `Chromap` also performs barcode correction and sorting, in addition to mapping a subset of the reads to the genome. Without the argument, `Chromap` only maps reads to the genome, but the entire read-set is mapped. The methods were benchmarked across multiple thread counts, with each process run three times per thread count, and the average across the three runs was computed to obtain the final metrics (total time taken and maximum memory used). The execution time metrics for the genomic read mapping process for `Chromap` (run using `--preset atac` argument), were obtained by subtracting the time taken for sorting, deduplication and writing from the total time taken for program execution. `Chromap` dumps the time for non-mapping related tasks to `stdout`. We ran `Cell Ranger ATAC` only once with 32 cores and 100 Gb of allowed RAM since it was not possible to benchmark the individual steps. `Cell Ranger ATAC` has previously been shown to be substantially slower than `Chromap`.

Software versions — The following software versions were used for the analyses in this manuscript: `Cell Ranger ATAC` version 2.1.0, `Chromap` version $0.2.7 - r493$, `Bowtie2` version 2.5.4, `mason` version 2.0.9, `MACS2` version 2.2.9.1, `R` version 4.3.2, `Signac` version 1.13.0, `tabix` version 1.16, and `Snakemake` version 8.18.0. Mapping for `alevin-fry-atac` was done using `piscem` version 0.11.0 and subsequent processing was done using the `alevin-fry-atac` branch of the `alevin-fry` repository.

Results

Mapping accuracy for `alevin-fry-atac`

We first varied the parameter ℓ_{vcol} (which controls the number of bases a virtual color will span, with most virtual colors spanning $\ell_{\mathrm{vcol}} + ov\_length$ bases), for a given k-mer size k and pseudoalignment threshold τ and observed its impact on mapping accuracy on the simulated data for the different read lengths (Figures S1 to S4). We find that accuracy decreases continuously as ℓ_{vcol} increases, irrespective of the values of the other parameters. Importantly, the sharpest decrease is observed when the chromosomes themselves are used as the colors for pseudoalignment (removing the effect of virtual colors on the procedure entirely). The accuracy drops to $84 - 86\%$ from being $92 - 96\%$ (depending on the read length) when using virtual colors. In general, we observe a drop in accuracy of $\sim 10\%$ or more when disabling virtual colors. Longer reads generally yield higher accuracy across most settings, except in the specific parameter configuration where $\tau = 1$ and virtual colors are disabled, where reads with length 100 have the highest accuracy.

We next varied the threshold τ while keeping ℓ_{vcol} and k fixed (Figures S5 to S8). The accuracy decreases as the threshold decreases from 0.6 to 1. The decline seems to be sharpest from 0.8 to 1, with the magnitude of decrease in accuracy in general being much higher for larger read lengths. For $\tau = 1$, at all the ℓ_{vcol} values, the accuracy of reads with length 150 is smaller than reads with length 100. However, when the chromosomes are used as colors, the accuracy for reads with length 150 increases when τ increases from $0.6 - 0.8$ and falls again at 1.

Finally, we varied k, while keeping $\ell_{\mathrm{vcol}}, \tau$ fixed (Figures S9 to S12). We observe that for reads of length 50, accuracy increases from $k = 23$ to $k = 25$ and starts decreasing across most virtual color extends and thresholds. However, when chromosomes are used as colors, accuracy increases with an increase in k. For the reads with larger lengths, a larger k is preferred as the accuracy increases uniformly across parameters.

Tables

Table S1. URLs for the references used in the manuscript for analysis.

Organism	URL
Homo Sapiens	`http://ftp.ensembl.org/pub/release-98/fasta/homo_sapiens/dna/Homo_sapiens.GRCh38.dna.primary_assembly.fa.gz`
Mus Musculus	`http://ftp.ensembl.org/pub/release-98/fasta/mus_musculus/dna/Mus_musculus.GRCm38.dna.primary_assembly.fa.gz`

Table S2. URLs for the datasets used in the manuscript for analysis.

Dataset	URL
`Human 10K PBMC`	10K
`Mouse 8K Cortex`	8k
`Human 3K Brain`	3K

Table S3. The total number of bases (MB) covered by the different methods across the different datasets. N/A entries denotes that `Cell Ranger ATAC` was not run on that dataset. `Cell Ranger ATAC` (`MACS2`) and `Cell Ranger ATAC` (Custom) denote that peaks were generated on the `Cell Ranger ATAC` fragments using `MACS2` and the inbuilt peak caller of `Cell Ranger ATAC` respectively.

Method/ Dataset	`alevin-fry-atac`	`Chromap`	`Cell Ranger ATAC` (`MACS2`)	`Cell Ranger ATAC` (Custom)
`Human 10K PBMC`	98.33	97.42	98.60	140.18
`Mouse 8K Cortex`	107.86	108.32	112.18	150.80
`Human 3K Brain`	115.14	111.80	N/A	N/A

Table S4. Percentage of overlap between the bases covered by the peaks unique to `Chromap` and `alevin-fry-atac`, when doing a pair-wise comparison with the bases spanning the soft-masked regions and the blacklist regions on the `Human 10K PBMC` dataset

Method/ Region	`alevin-fry-atac`	`Chromap`
Soft-masked	47.85	55.08
Blacklist	1.15	1.49

Figures

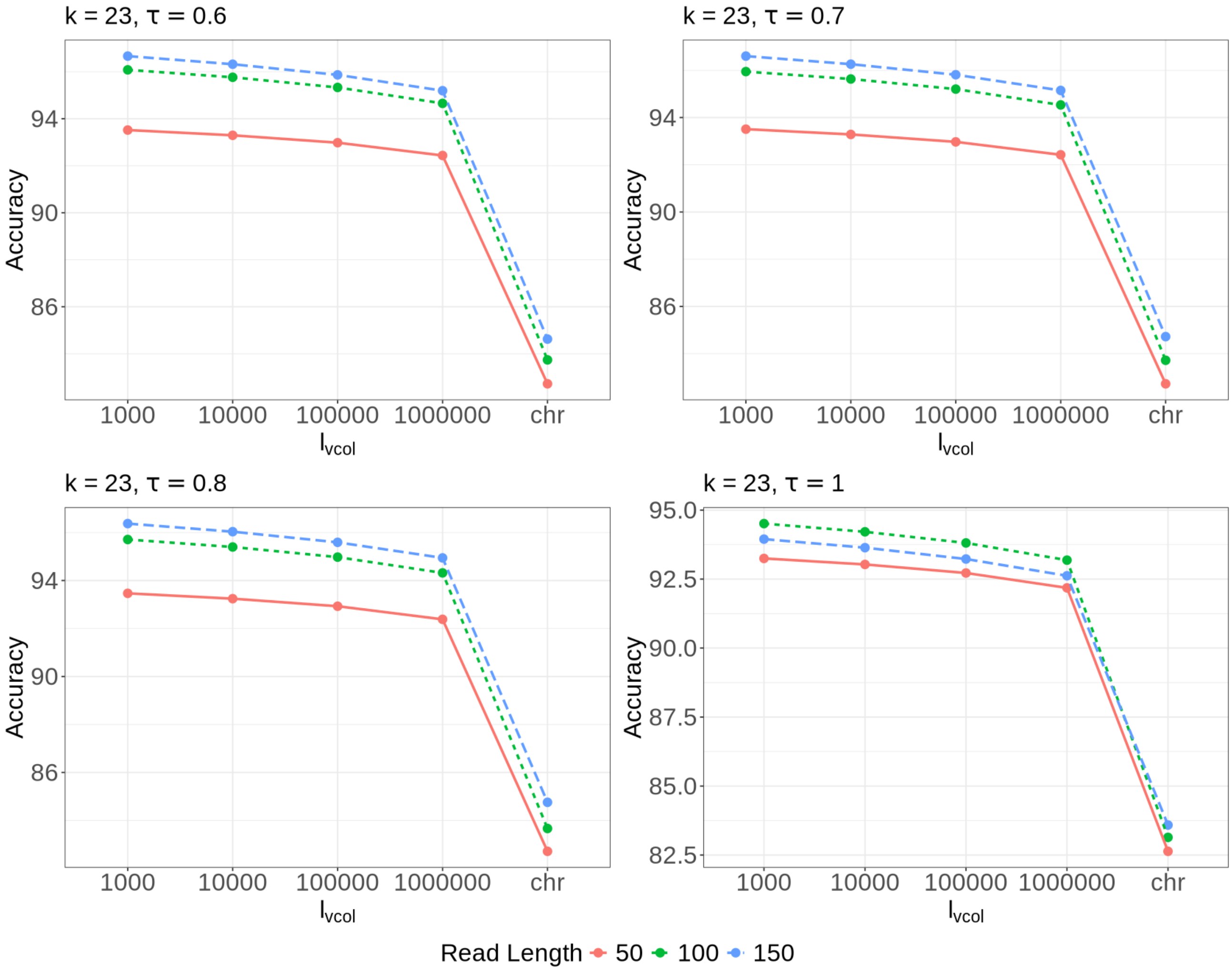

Fig. S1. Evaluating accuracy across different read lengths for `alevin-fry-atac` by varying ℓ_{vcol} at $k = 23$, with panels representing different τ values. The label *chr* on the x-axis implies that mapping is done using chromosome sequence as colors (removing the impact of virtual colors on the mapping procedure).

References

1. HM Amemiya, A Kundaje, and AP Boyle. The encode blacklist: identification of problematic regions of the genome. sci. rep. 9, 9354, 2019.
2. Alexander Dobin, Carrie A Davis, Felix Schlesinger, Jorg Drenkow, Chris Zaleski, Sonali Jha, Philippe Batut, Mark Chaisson, and Thomas R Gingeras. STAR: ultrafast universal RNA-seq aligner. *Bioinformatics*, 29(1):15–21, 2013.
3. John M. Gaspar. Improved peak-calling with MACS2. *bioRxiv*, 2018.
4. Felix Mölder, Kim Philipp Jablonski, Brice Letcher, Michael B Hall, Christopher H Tomkins-Tinch, Vanessa Sochat, Jan Forster, Soohyun Lee, Sven O Twardziok, Alexander Kanitz, et al. Sustainable data analysis with Snakemake. *F1000Research*, 10, 2021.
5. Kristoffer Sahlin. Effective sequence similarity detection with strobemers. *Genome research*, 31(11):2080–2094, 2021.
6. Tim Stuart, Avi Srivastava, Shaista Madad, Caleb A Lareau, and Rahul Satija. Single-cell chromatin state analysis with Signac. *Nature methods*, 18(11):1333–1341, 2021.
7. Andrew D Yates, Premanand Achuthan, Wasiu Akanni, James Allen, Jamie Allen, Jorge Alvarez-Jarreta, M Ridwan Amode, Irina M Armean, Andrey G Azov, Ruth Bennett, et al. Ensembl 2020. *Nucleic acids research*, 48(D1):D682–D688, 2020.
8. Haowen Zhang, Li Song, Xiaotao Wang, Haoyu Cheng, Chenfei Wang, Clifford A Meyer, Tao Liu, Ming Tang, Srinivas Aluru, Feng Yue, et al. Fast alignment and preprocessing of chromatin profiles with Chromap. *Nature communications*, 12(1):6566, 2021.

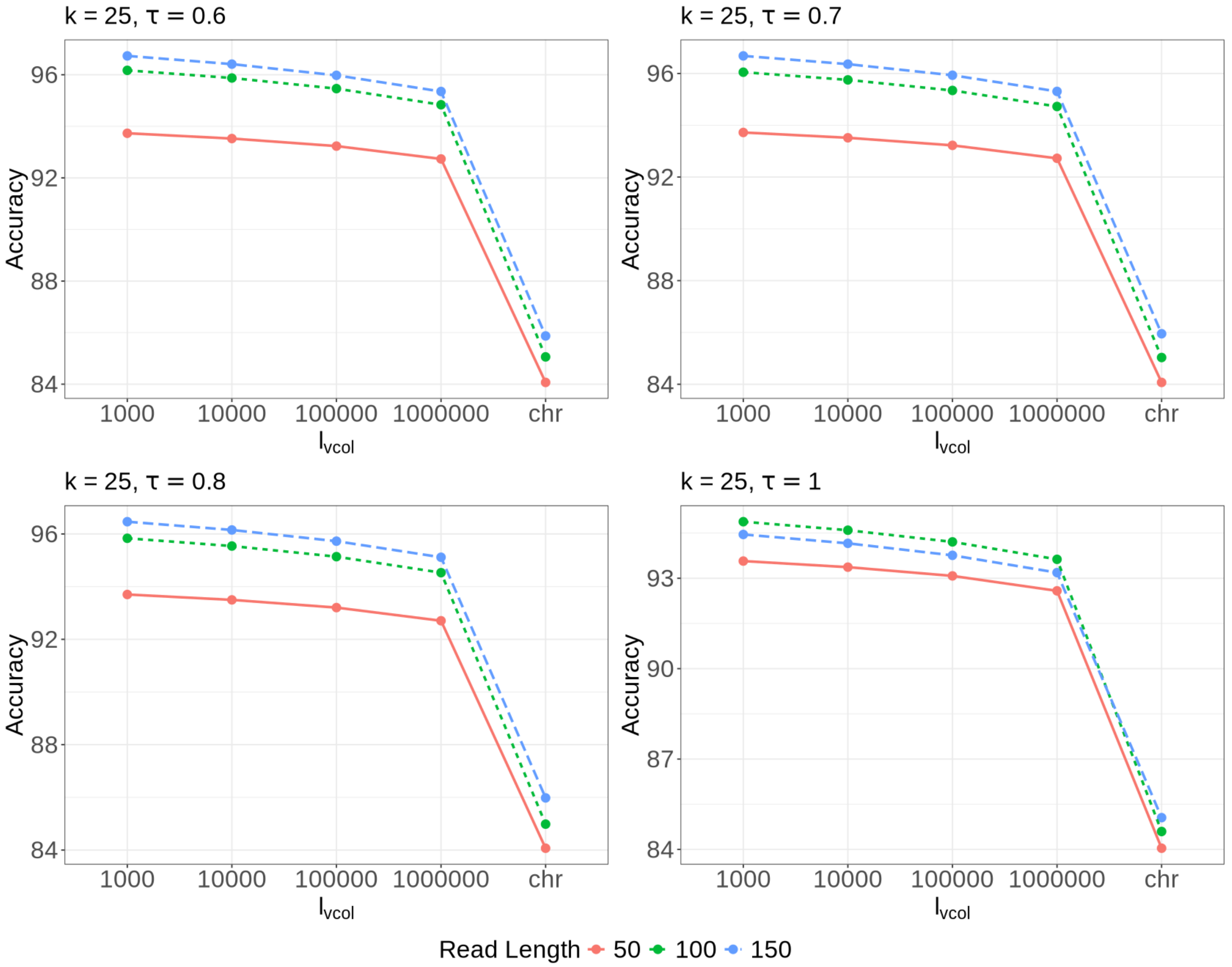

Fig. S2. Evaluating accuracy across different read lengths for `alevin-fry-atac` by varying ℓ_{vcol} at $k = 25$, with panels representing different τ values. The label *chr* on the x-axis implies that mapping is done using chromosome sequence as colors (removing the impact of virtual colors on the mapping procedure).

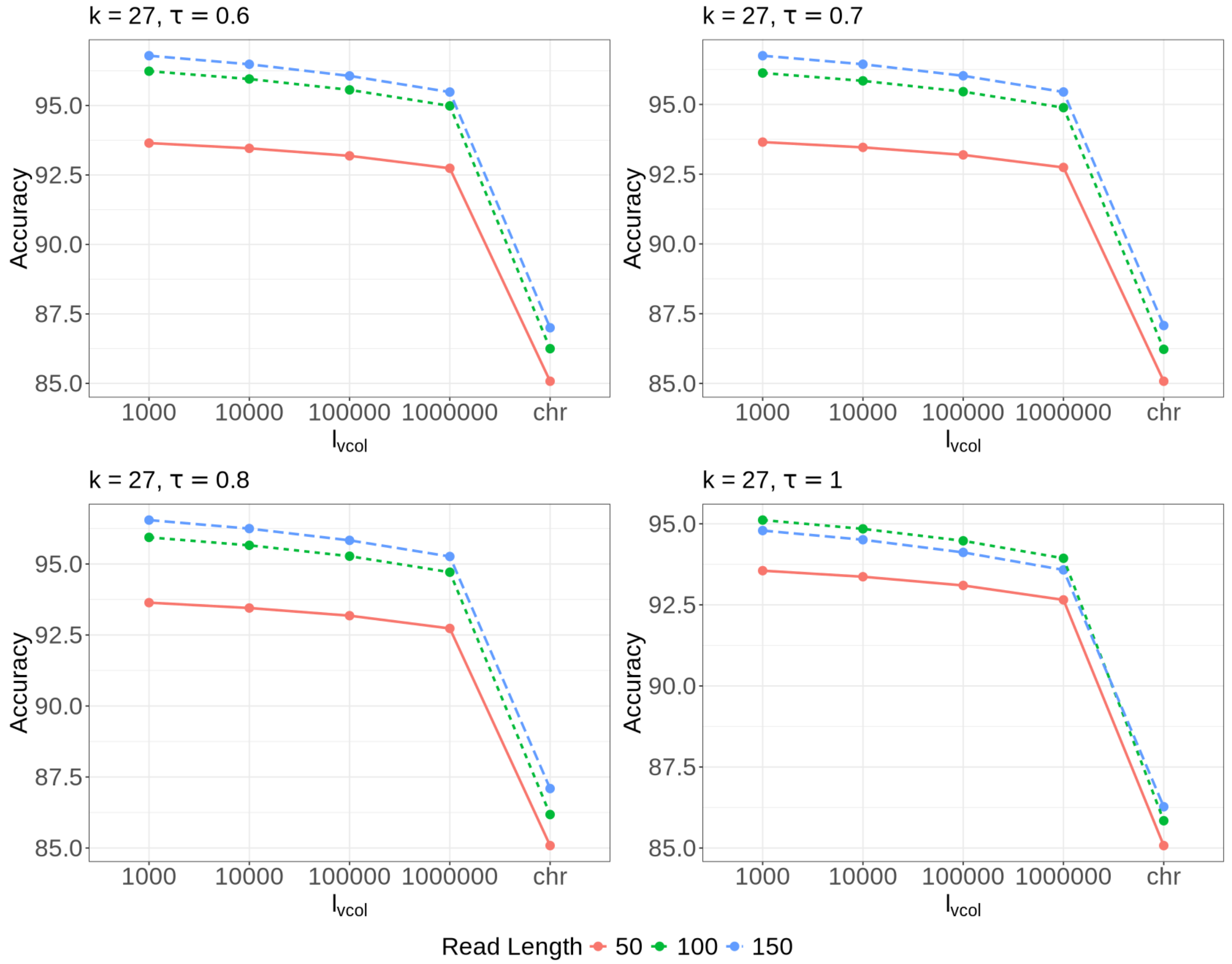

Fig. S3. Evaluating accuracy across different read lengths for `alevin-fry-atac` by varying ℓ_{vcol} for $k = 27$ with panels representing different τ values. The label *chr* on the x-axis implies that mapping is done using chromosome sequence as colors (removing the impact of virtual colors on the mapping procedure).

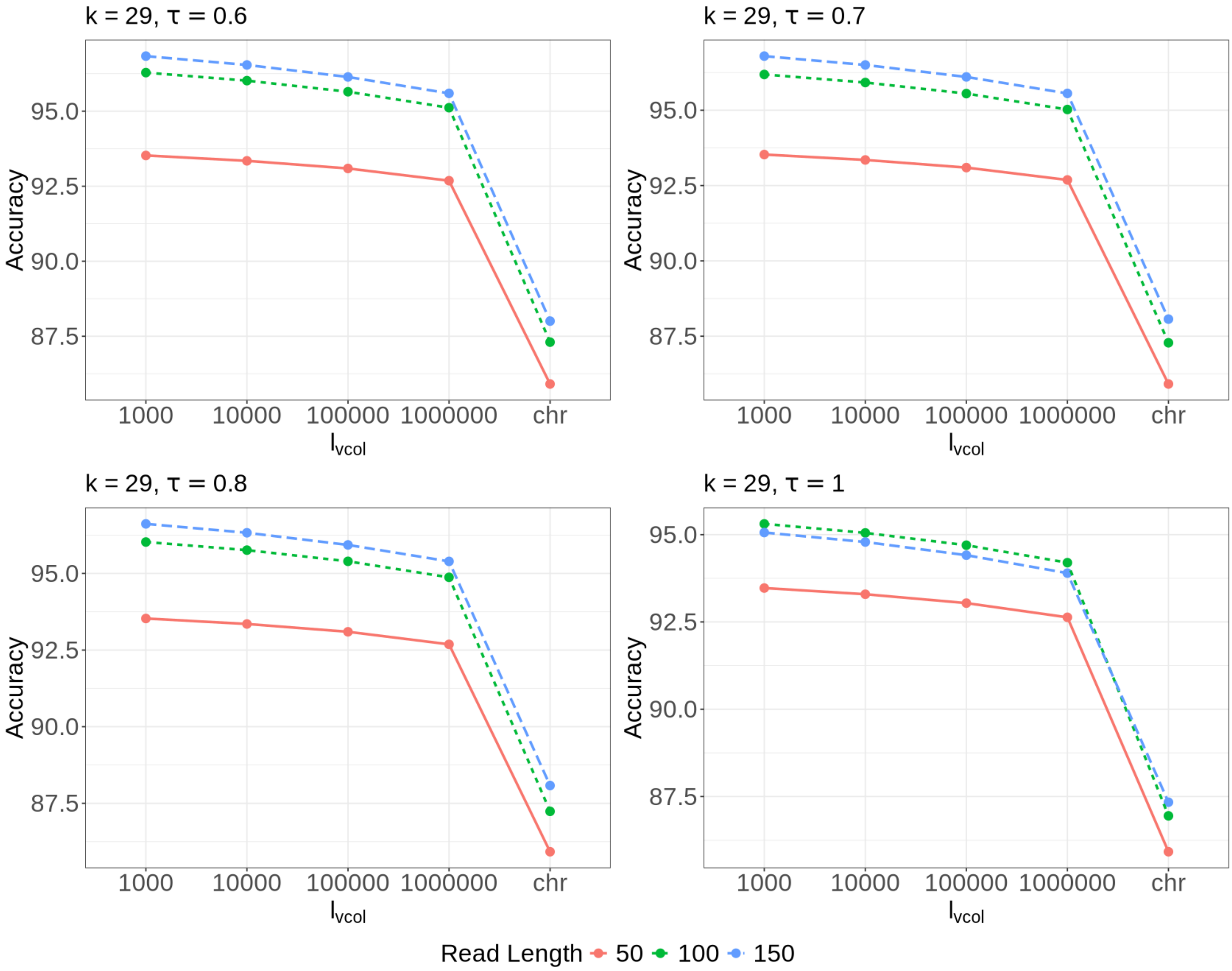

Fig. S4. Evaluating accuracy across different read lengths for `alevin-fry-atac` by varying ℓ_{vcol} for $k = 29$ with panels representing different τ values. The label *chr* on the x-axis implies that mapping is done using chromosome sequence as colors (removing the impact of virtual colors on the mapping procedure).

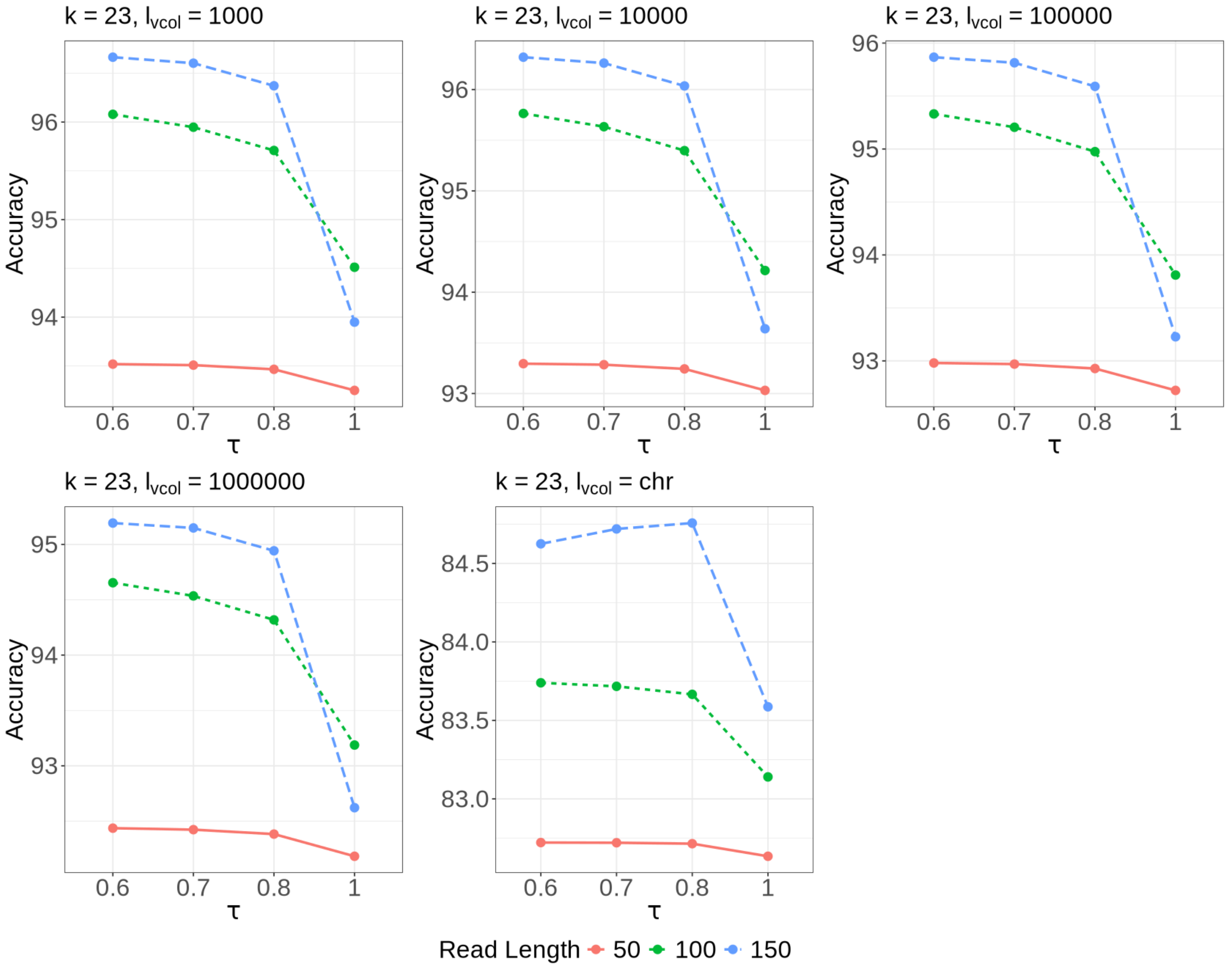

Fig. S5. Evaluating accuracy across different read lengths for `alevin-fry-atac` by varying the pseudoalignment thresholds for $k = 23$ with panels representing different ℓ_{vcol} values. The panel with ℓ_{vcol} lchr implies that mapping is done using chromosome sequence as colors (removing the impact of virtual colors on the mapping procedure).

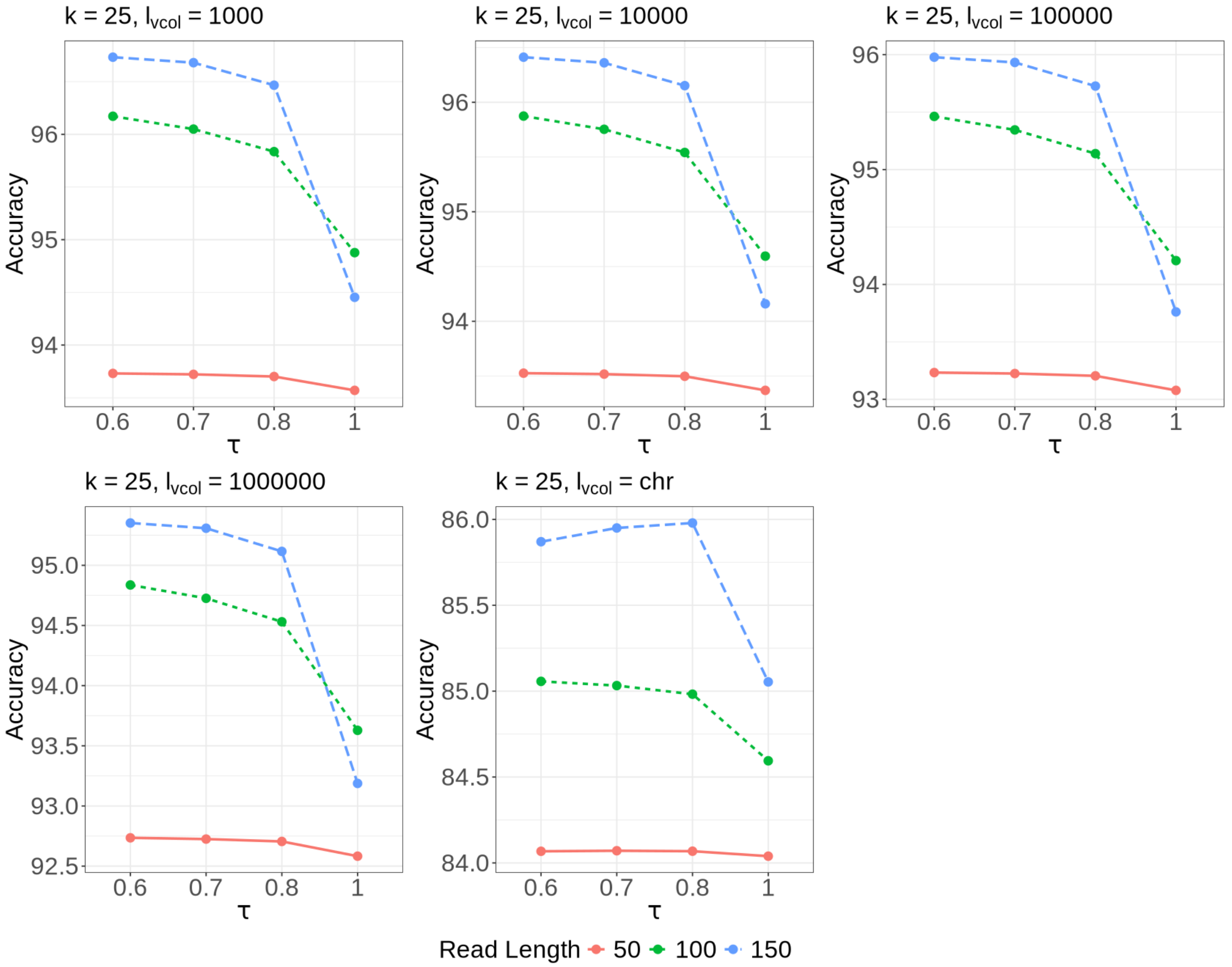

Fig. S6. Evaluating accuracy across different read lengths for `alevin-fry-atac` by varying the pseudoalignment thresholds for $k = 25$ with panels representing different ℓ_{vcol} values. The panel with ℓ_{vcol} *chr* implies that mapping is done using chromosome sequence as colors (removing the impact of virtual colors on the mapping procedure).

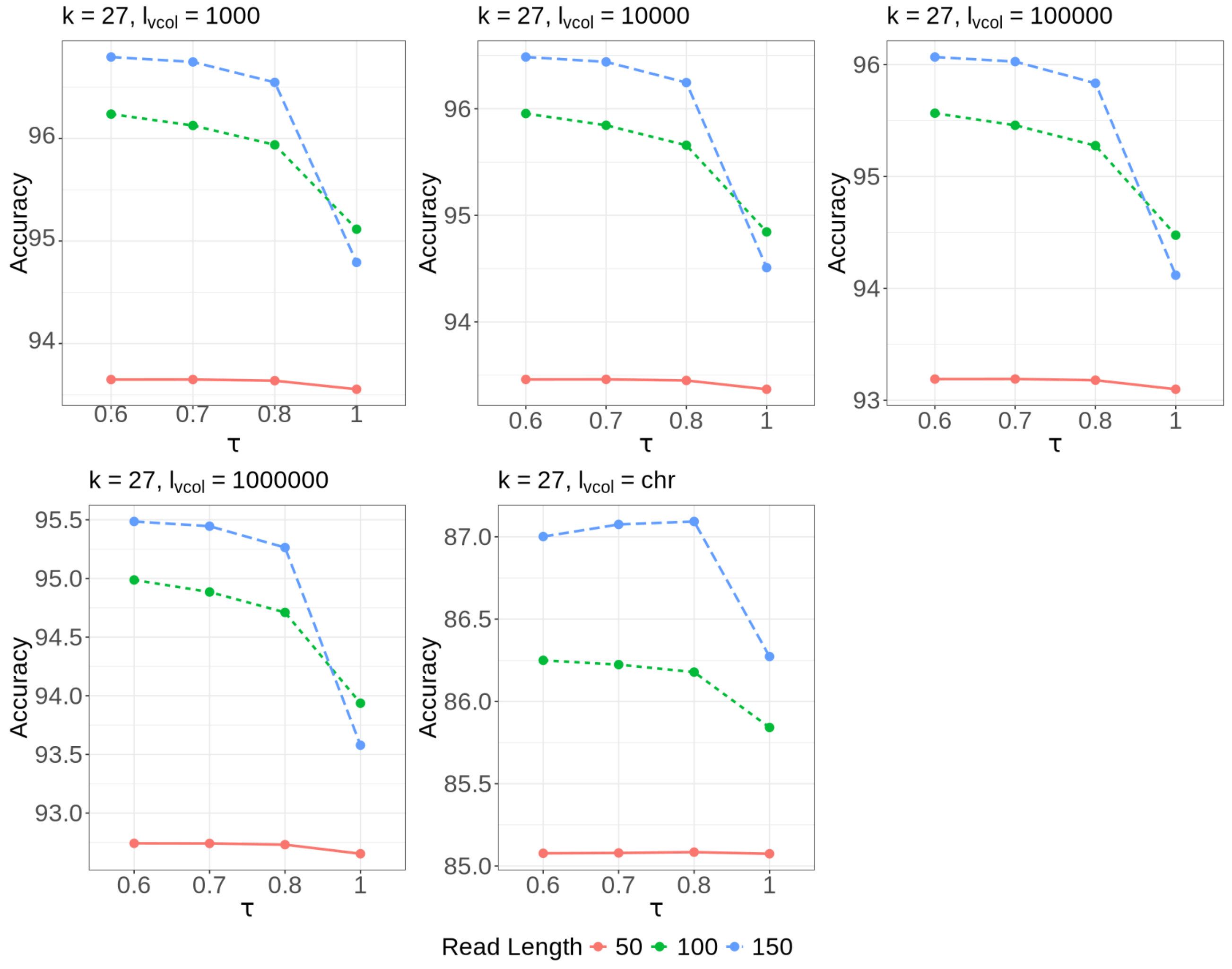

Fig. S7. Evaluating accuracy across different read lengths for `alevin-fry-atac` by varying the pseudoalignment thresholds for $k = 27$ with panels representing different ℓ_{vcol} values. The panel with ℓ_{vcol} *chr* implies that mapping is done using chromosome sequence as colors (removing the impact of virtual colors on the mapping procedure).

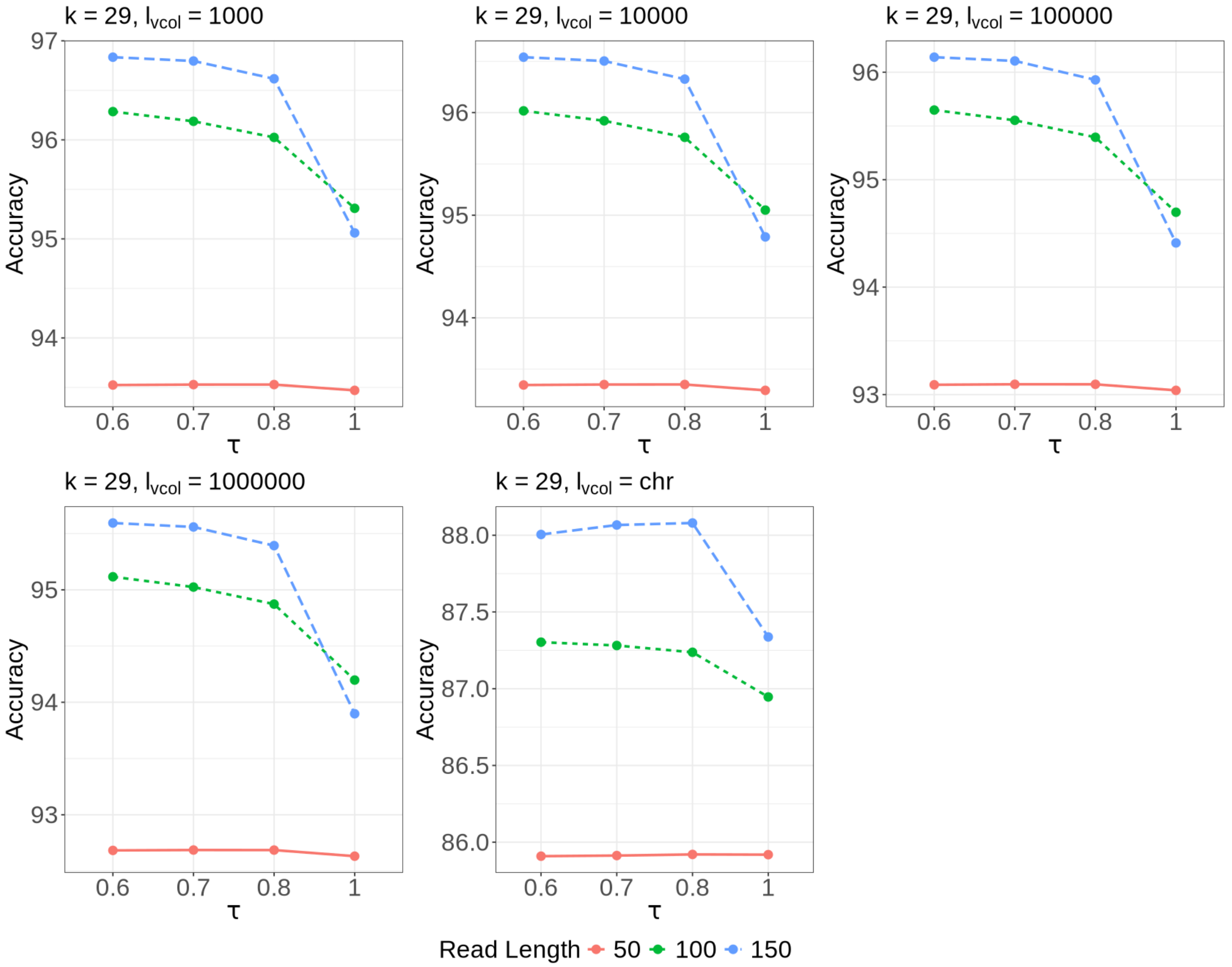

Fig. S8. Evaluating accuracy across different read lengths for `alevin-fry-atac` by varying the pseudoalignment thresholds for $k = 29$ with panels representing different ℓ_{vcol} values. The panel with bin length *chr* implies that mapping is done using chromosome sequence as colors (removing the impact of virtual colors on the mapping procedure).

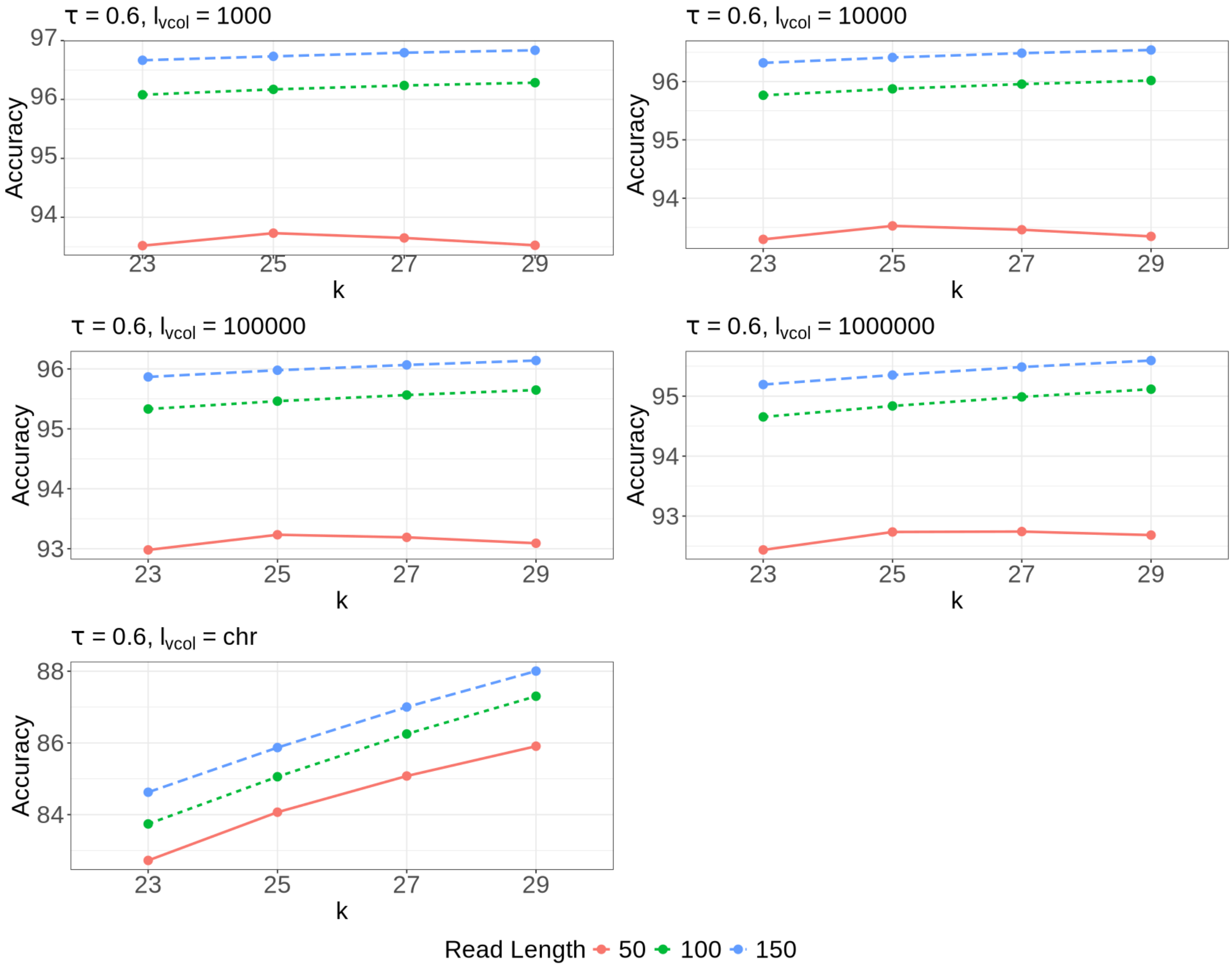

Fig. S9. Evaluating accuracy across different read lengths for `alevin-fry-atac` by varying the k-mer size for $\tau = 0.6$ with panels representing different ℓ_{vcol} values. The panel with ℓ_{vcol} *chr* implies that mapping is done using chromosome sequence as colors (removing the impact of virtual colors on the mapping procedure).

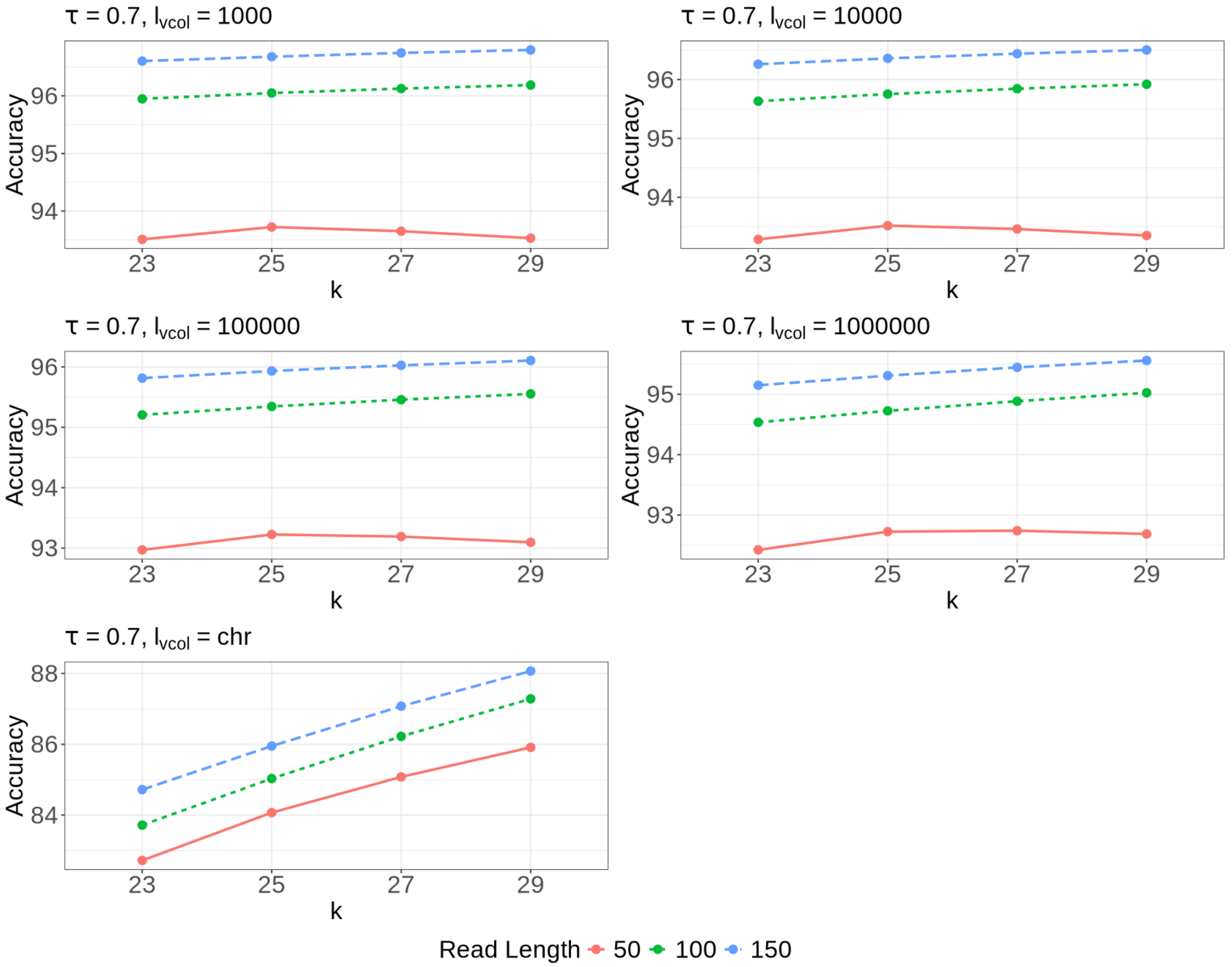

Fig. S10. Evaluating accuracy across different read lengths for `alevin-fry-atac` by varying the k-mer size for $\tau = 0.7$ with panels representing different ℓ_{vcol} values. The panel with ℓ_{vcol} *chr* implies that mapping is done using chromosome sequence as colors (removing the impact of virtual colors on the mapping procedure).

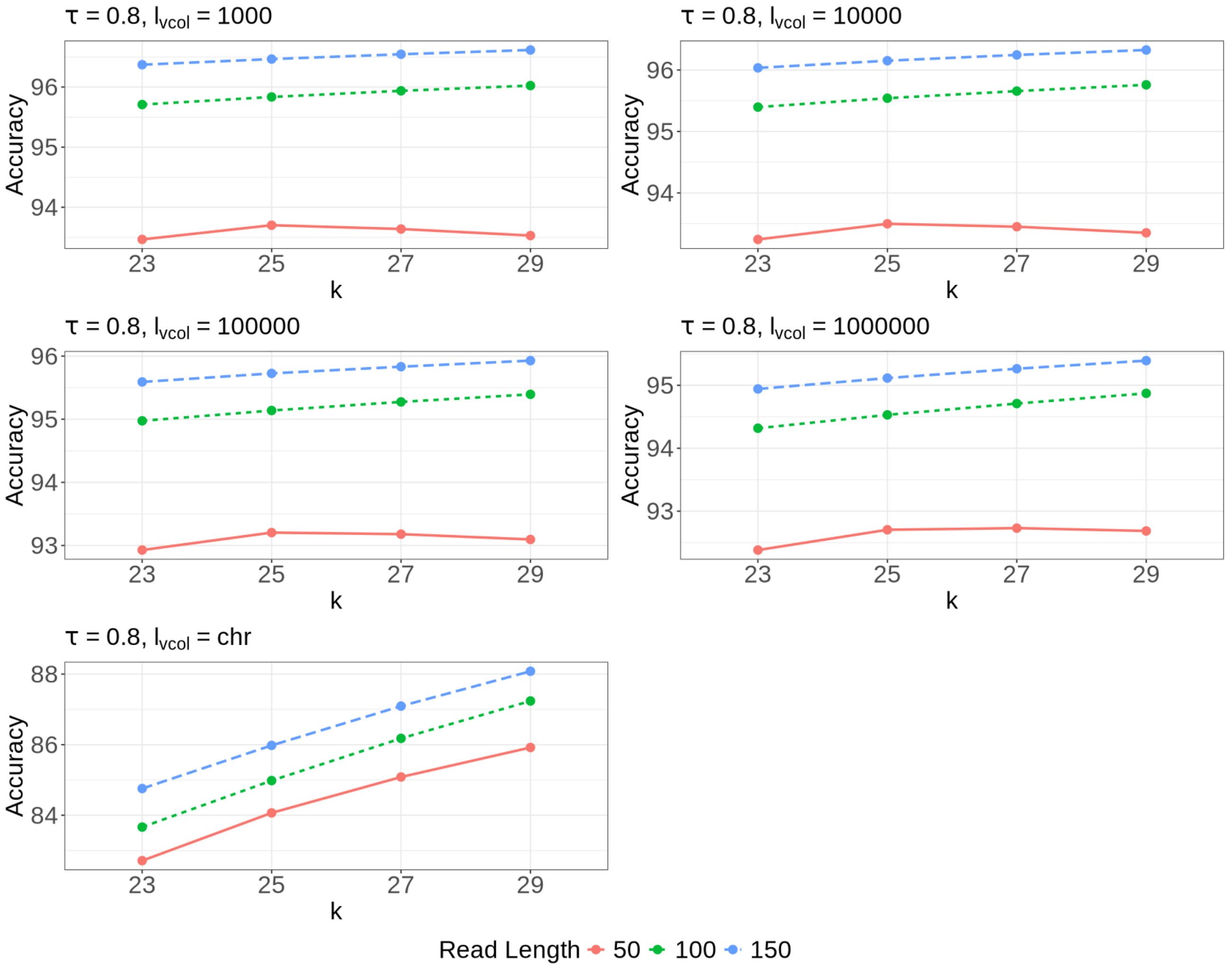

Fig. S11. Evaluating accuracy across different read lengths for `alevin-fry-atac` by varying the k-mer size for $\tau = 0.8$ with panels representing different ℓ_{vcol} values. The panel with ℓ_{vcol} *chr* implies that mapping is done using chromosome sequence as colors (removing the impact of virtual colors on the mapping procedure).

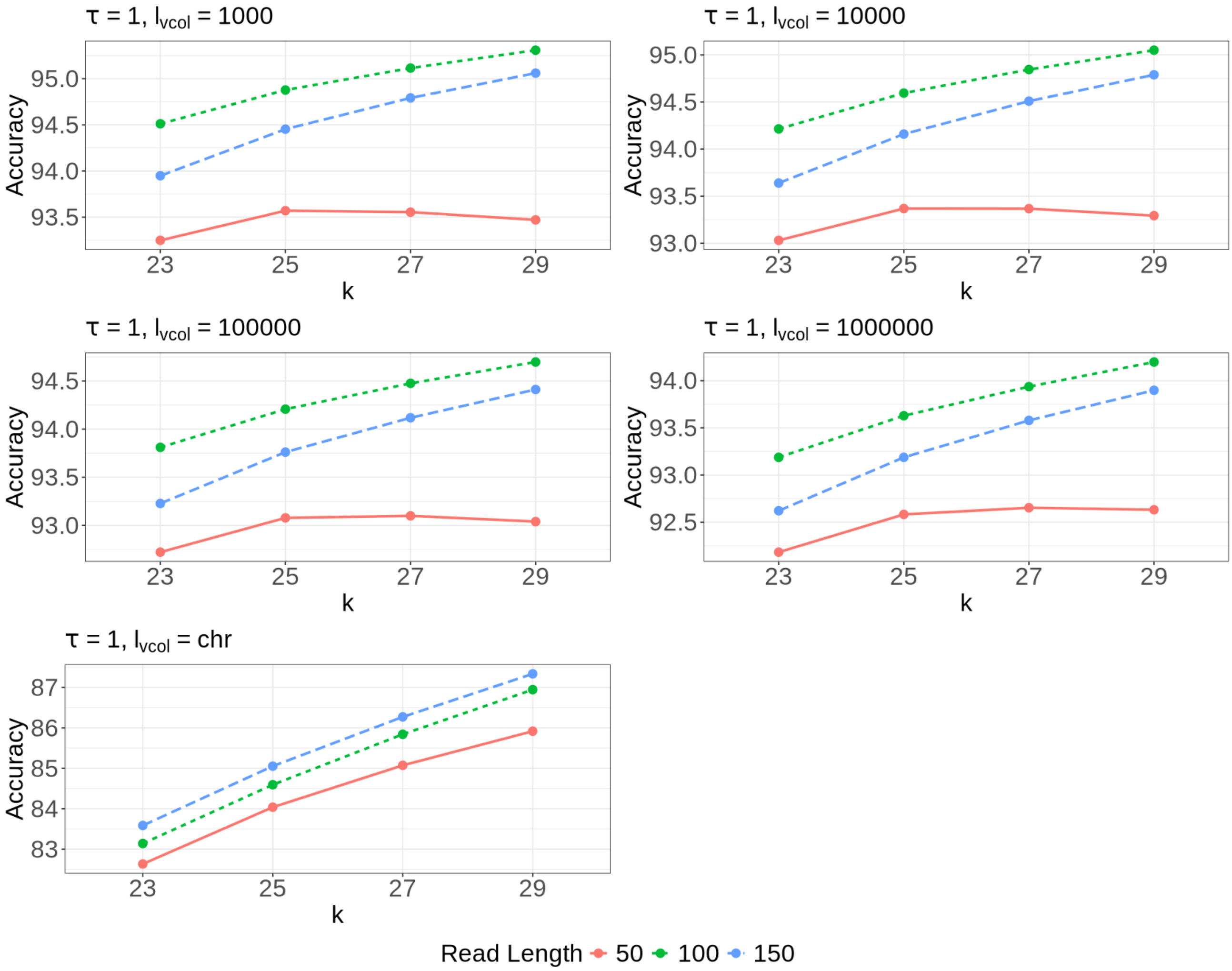

Fig. S12. Evaluating accuracy across different read lengths for `alevin-fry-atac` by varying the k-mer size for $\tau = 1$ with panels representing different ℓ_{vcol} values. The panel with ℓ_{vcol} *chr* implies that mapping is done using chromosome sequence as colors (removing the impact of virtual colors on the mapping procedure).

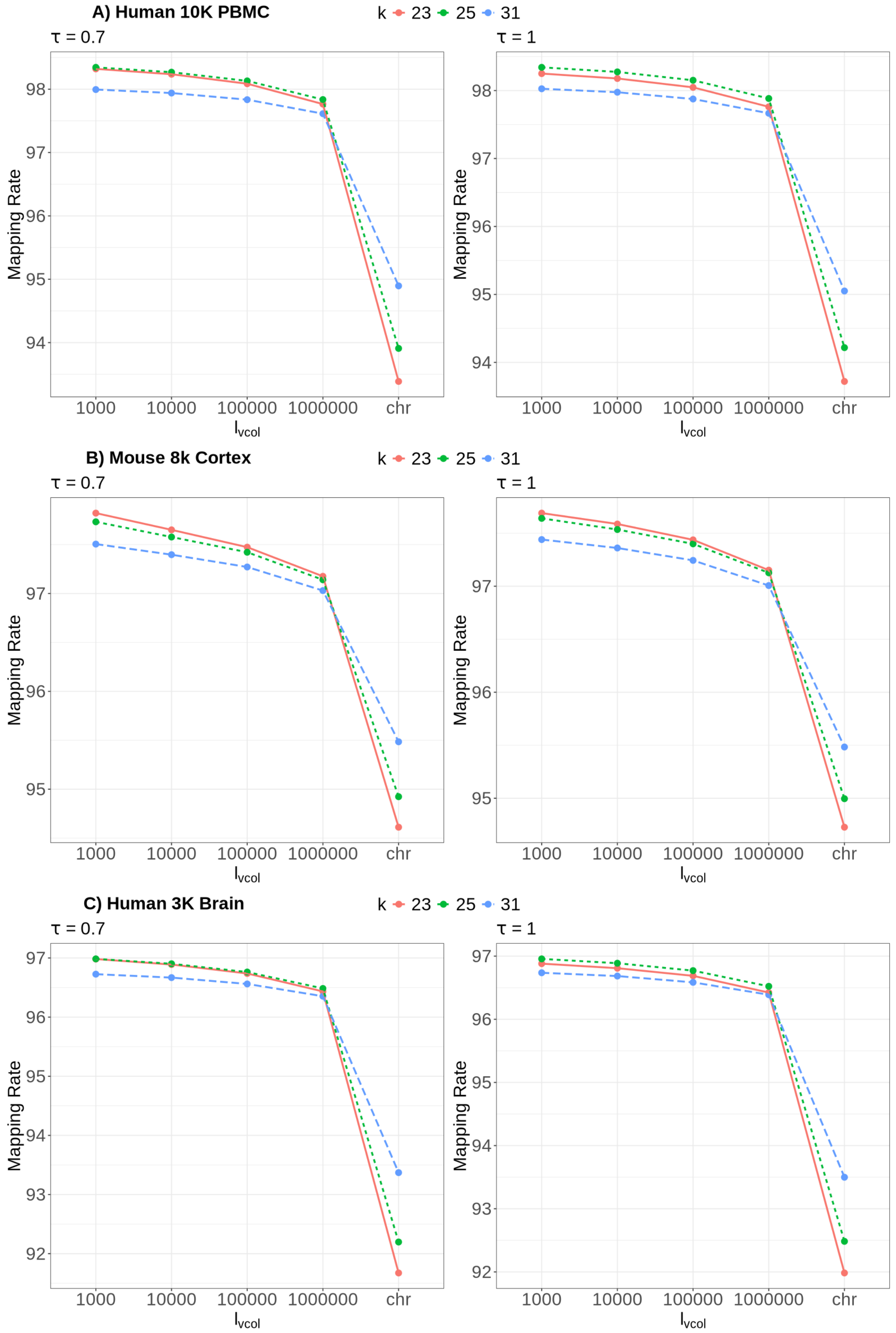

Fig. S13. Evaluating the mapping rate across different k-mer size for `alevin-fry-atac` by varying ℓ_{vcol} for A) $\tau = 0.7$, B) $\tau = 1$ on the `Human 10K PBMC`, `Mouse 8K Cortex` and `Human 3K Brain` datasets respectively. The x-axis labels *chr* implies mapping is done using chromosome sequence as colors (removing the impact of virtual colors on mapping procedure).

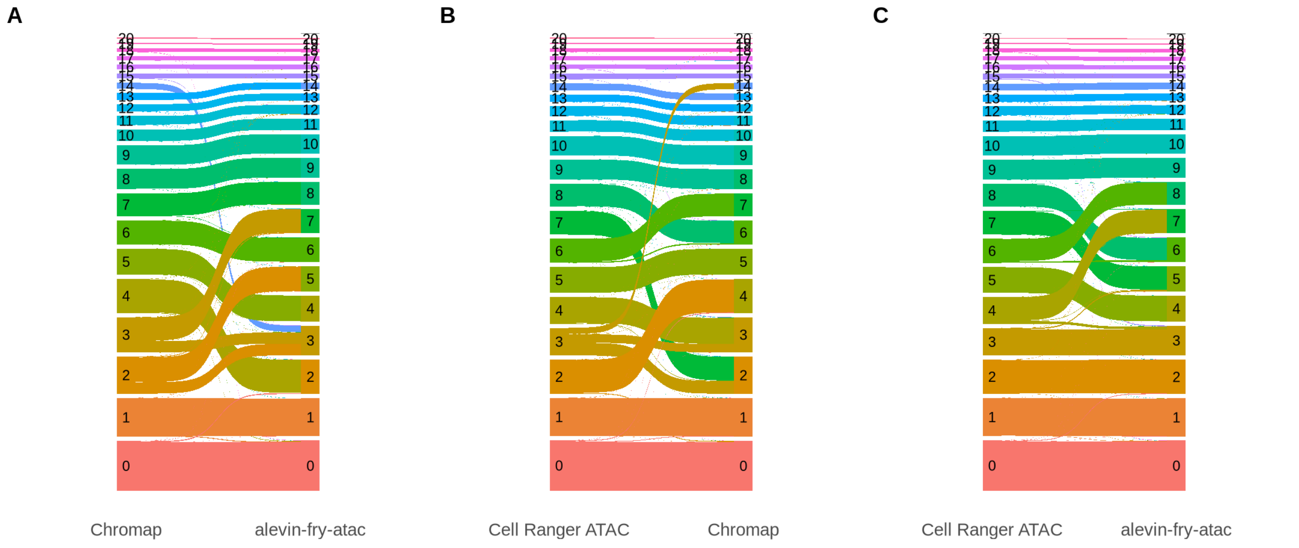

Fig. S14. Pairwise Sankey plot on the clusters obtained across the different methods for `Human 10K PBMC` dataset.

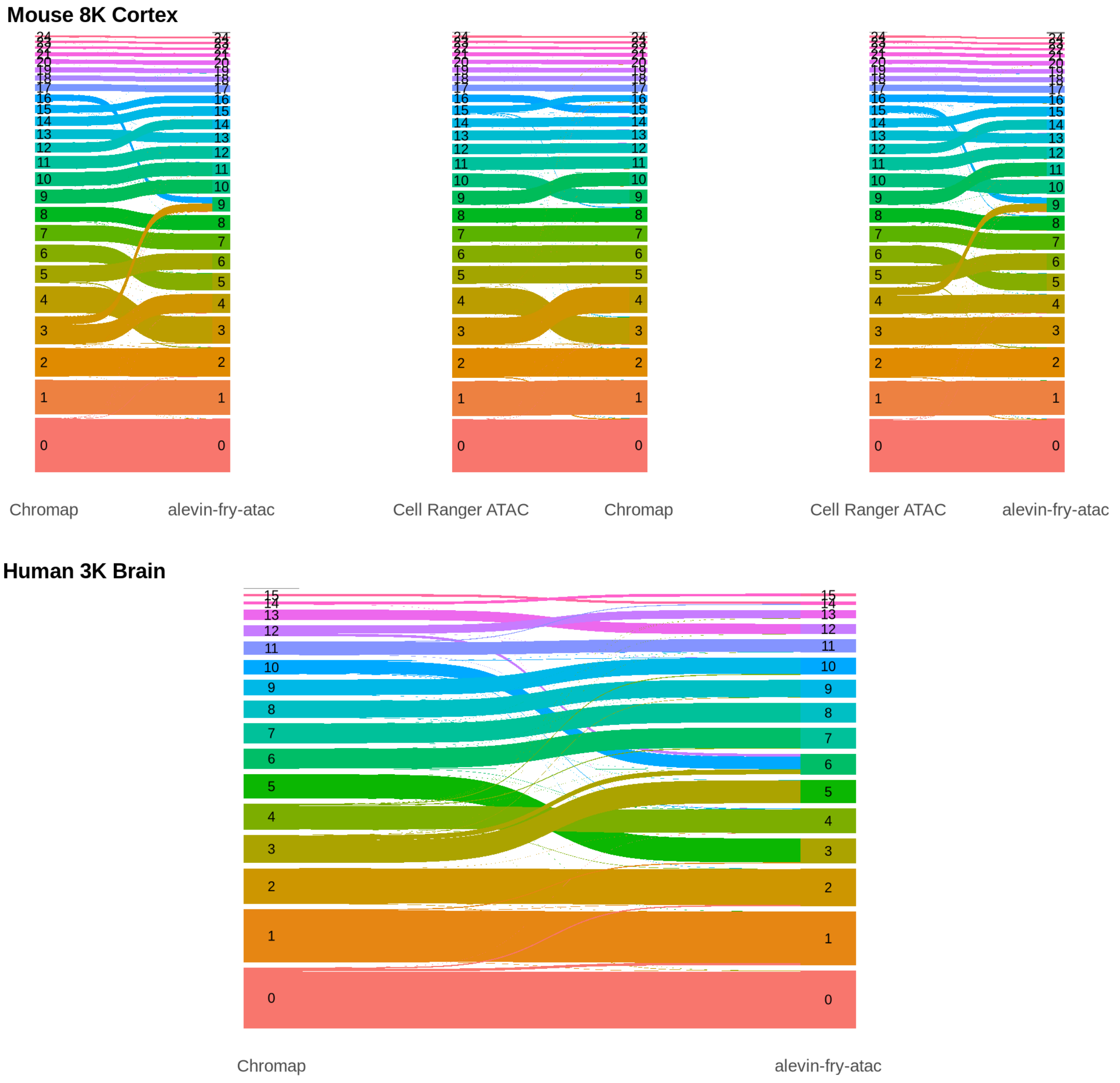

Fig. S15. Pairwise Sankey plot on the clusters obtained across the different methods for `Mouse 8K Cortex` and `Human 3K Brain` dataset.

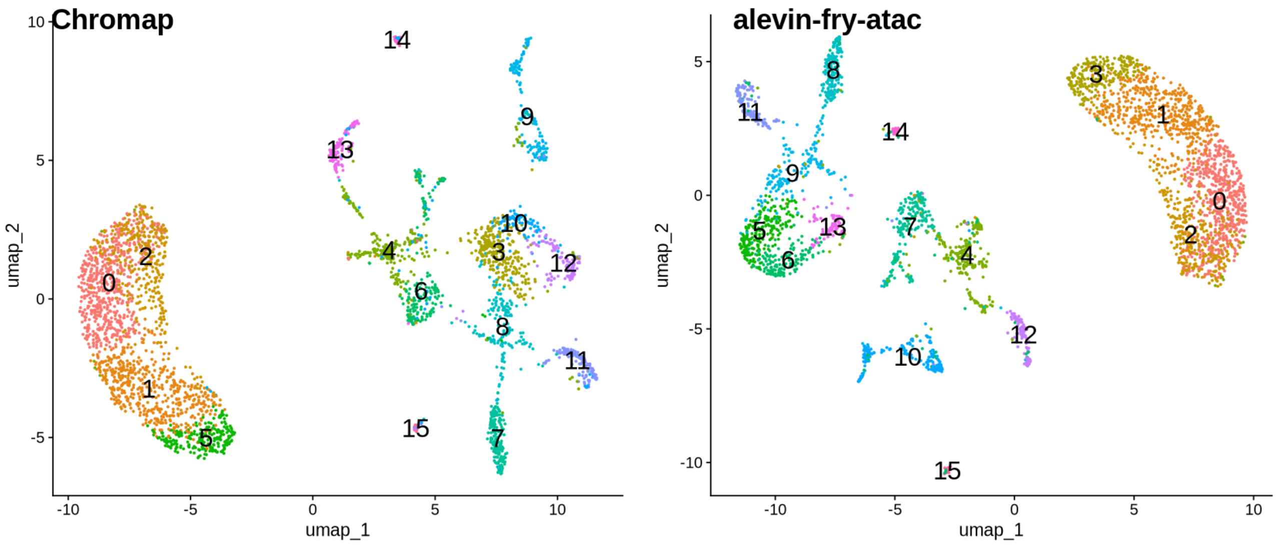

Fig. S16. UMAP projections with the cluster labels obtained for the different methods on the `Human 10K PBMC` dataset.

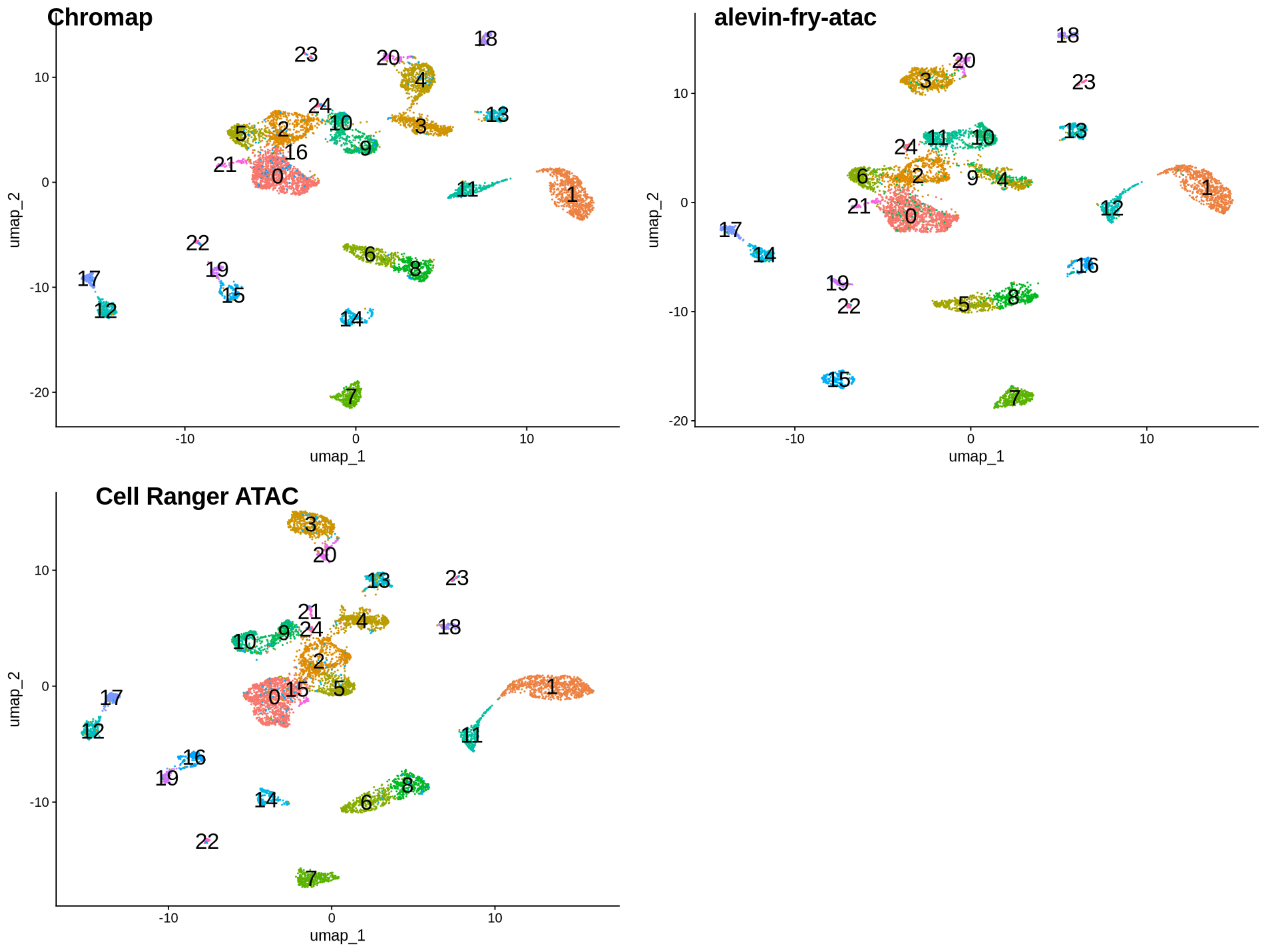

Fig. S17. UMAP projections with the cluster labels obtained for the different methods on the `Mouse 8K Cortex` dataset.

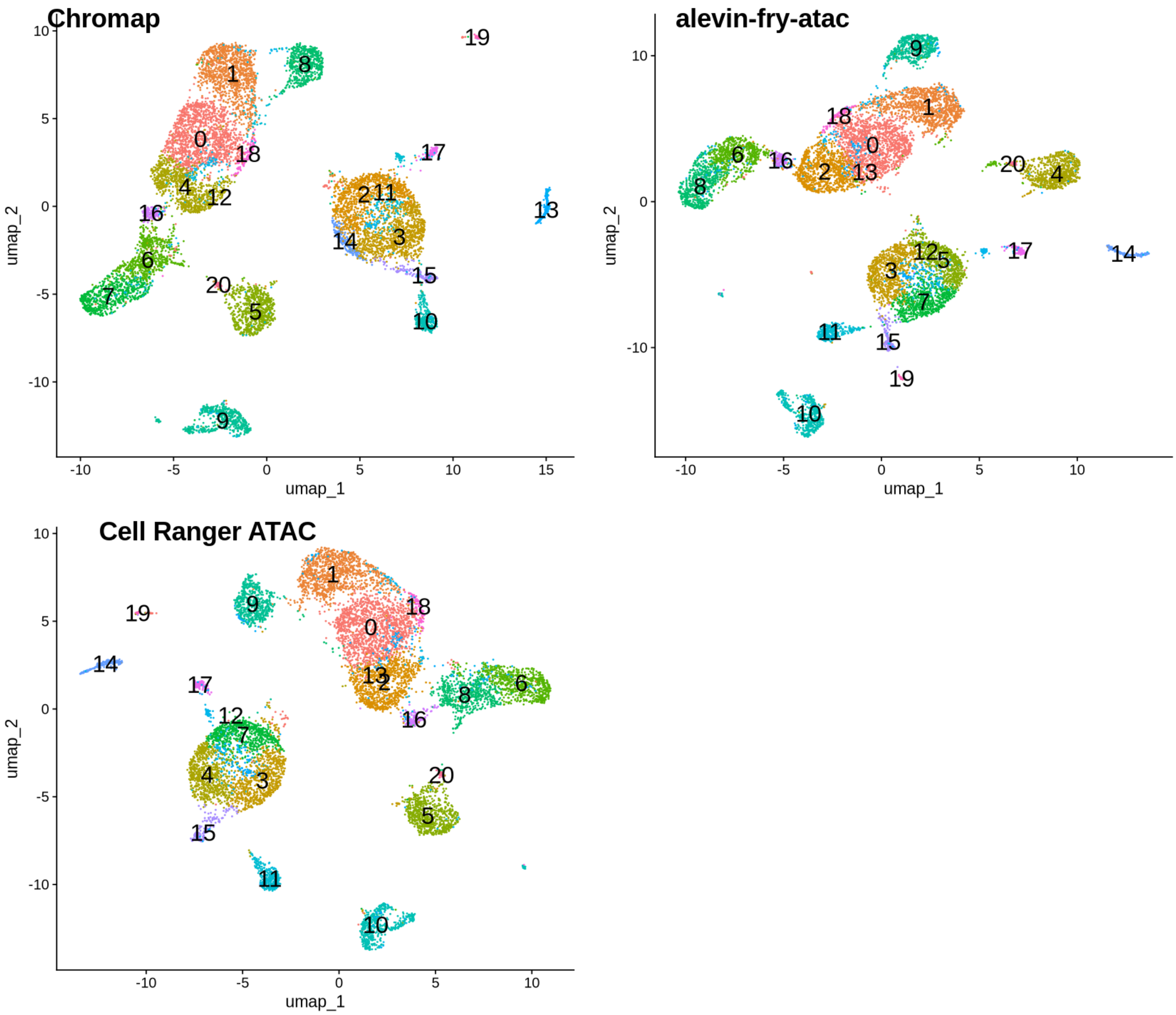

Fig. S18. UMAP projections with the cluster labels obtained for the different methods on the `Human 3K Brain` dataset.

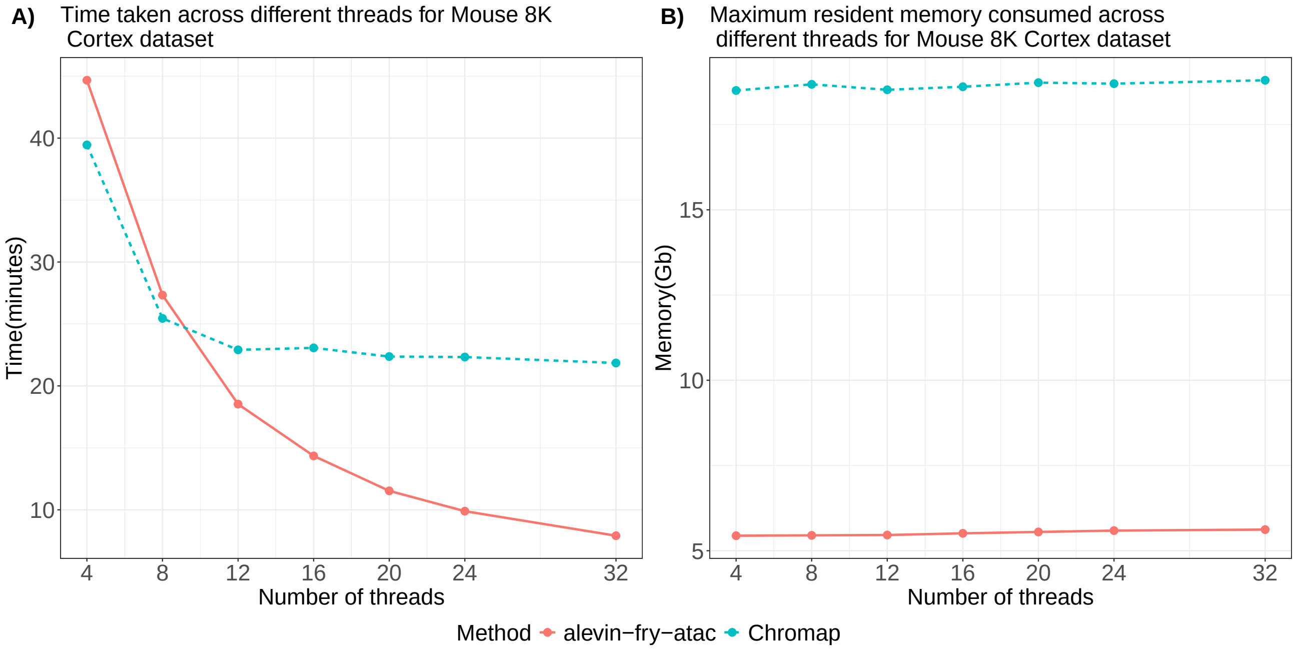

Fig. S19. Comparing the time taken and maximum resident memory consumed by `alevin-fry-atac` and `Chromap` for the `Mouse 8K Cortex` dataset across the different threads.

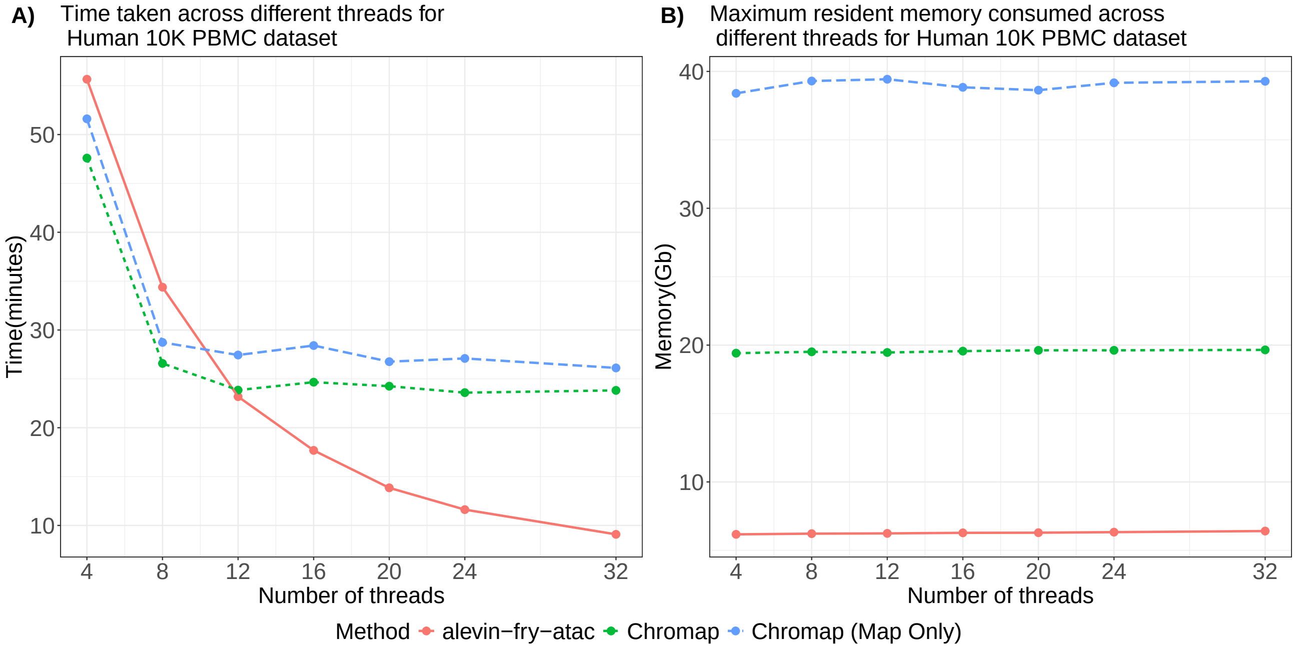

Fig. S20. Comparing the time taken and maximum resident memory consumed by `alevin-fry-atac` and `Chromap` for the `Human 10K PBMC` dataset across the different threads to map the reads. `Chromap` (Map Only) refers to when `Chromap` is run wihout the `--preset` arguments.

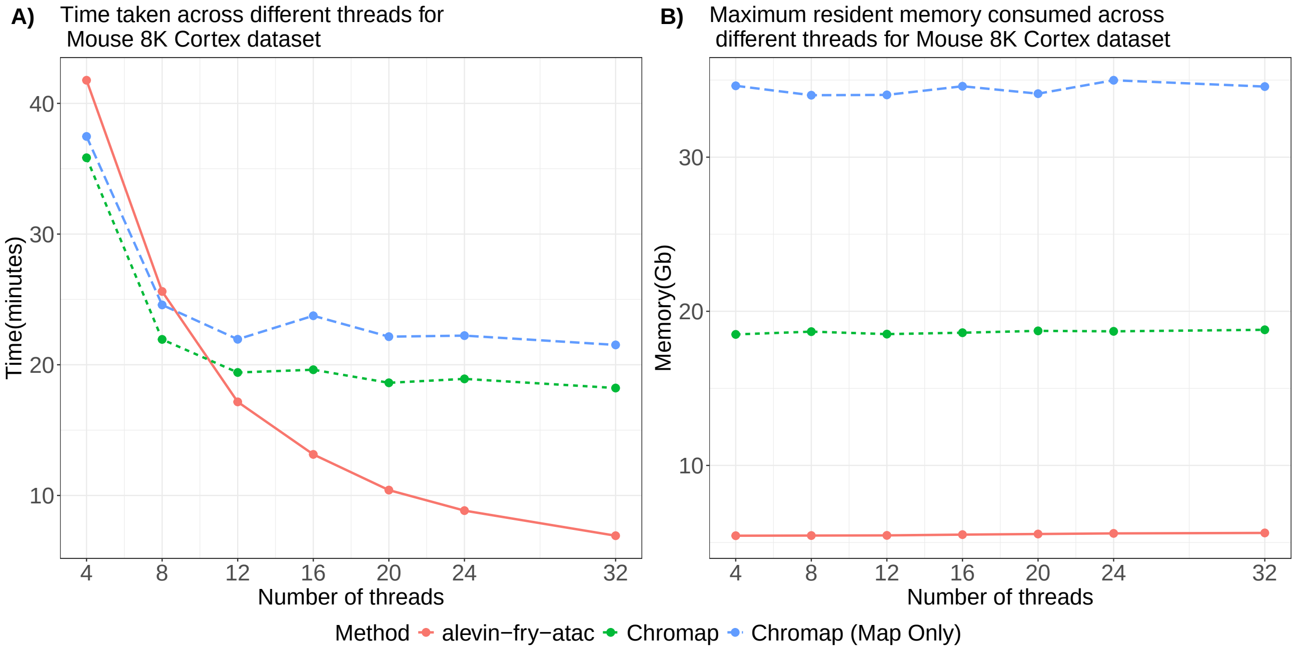

Fig. S21. Comparing the time taken and maximum resident memory consumed by `alevin-fry-atac` and `Chromap` for the `Mouse 8K Cortex` dataset across the different threads to map the reads. `Chromap` (Map Only) refers to when `Chromap` is run wihout the `--preset` arguments.
